# Microbiome composition and geochemical characteristics of deep subsurface high-pressure environment, Pyhäsalmi mine Finland

**DOI:** 10.3389/fmicb.2015.01203

**Published:** 2015-10-30

**Authors:** Hanna Miettinen, Riikka Kietäväinen, Elina Sohlberg, Mikko Numminen, Lasse Ahonen, Merja Itävaara

**Affiliations:** ^1^Valtion Teknillinen Tutkimuskeskus Technical Research Centre of Finland Ltd.Espoo, Finland; ^2^Geological Survey of FinlandEspoo, Finland; ^3^Pyhäsalmi Mine Oy, First Quantum Minerals Ltd.Pyhäsalmi, Finland

**Keywords:** microbiome, geochemical characterization, crystalline bedrock, gas, deep subsurface, high-pressure sampling

## Abstract

Pyhäsalmi mine in central Finland provides an excellent opportunity to study microbial and geochemical processes in a deep subsurface crystalline rock environment through near-vertical drill holes that reach to a depth of more than two kilometers below the surface. However, microbial sampling was challenging in this high-pressure environment. Nucleic acid yields obtained were extremely low when compared to the cell counts detected (1.4 × 10^4^ cells mL^−1^) in water. The water for nucleic acid analysis went through high decompression (60–130 bar) during sampling, whereas water samples for detection of cell counts by microscopy could be collected with slow decompression. No clear cells could be identified in water that went through high decompression. The high-pressure decompression may have damaged part of the cells and the nucleic acids escaped through the filter. The microbial diversity was analyzed from two drill holes by pyrosequencing amplicons of the bacterial and archaeal 16S rRNA genes and from the fungal ITS regions from both DNA and RNA fractions. The identified prokaryotic diversity was low, dominated by Firmicute, Beta- and Gammaproteobacteria species that are common in deep subsurface environments. The archaeal diversity consisted mainly of Methanobacteriales. Ascomycota dominated the fungal diversity and fungi were discovered to be active and to produce ribosomes in the deep oligotrophic biosphere. The deep fluids from the Pyhäsalmi mine shared several features with other deep Precambrian continental subsurface environments including saline, Ca-dominated water and stable isotope compositions positioning left from the meteoric water line. The dissolved gas phase was dominated by nitrogen but the gas composition clearly differed from that of atmospheric air. Despite carbon-poor conditions indicated by the lack of carbon-rich fracture fillings and only minor amounts of dissolved carbon detected in formation waters, some methane was found in the drill holes. No dramatic differences in gas compositions were observed between different gas sampling methods tested. For simple characterization of gas composition the most convenient way to collect samples is from free flowing fluid. However, compared to a pressurized method a relative decrease in the least soluble gases may appear.

## Introduction

Microbial life has been observed in deep subsurface continental bedrock fractures and aquifers despite the harsh conditions in terms of energy, nutrients, pressure, and space. Nutrient and energy sources in the form of dissolved gases, solutes, or colloids are available via fracture fluids as most crystalline rocks lack extensive and continuous water-filled porosity. In a fractured rock environment, rock itself may provide a nutritional and energetic substrate to sustain minimal metabolic activity (Fredrickson and Balkwill, 2006). Microbial cells in these settings catabolize 10^4^–10^6^ fold more slowly than model organisms in nutrient-rich cultures and subsist with energy fluxes that are 1000-fold lower than the typical culture-based estimates of maintenance requirements (Hoehler and Jørgensen, 2013). The total number of bacteria found in groundwater ecosystems may vary by several orders of magnitude between 10^2^ and 10^6^ cells per mL of groundwater (Griebler and Lueders, 2009; Itävaara et al., 2011; Nyyssönen et al., 2012). The dominant bacterial and archaeal groups in different deep subsurface bedrock environments vary based on prevailing lithology and hydrogeochemistry (Nyyssönen et al., 2014; Bomberg et al., 2015). The diverse microbial communities detected in deep subsurface exploit various metabolic pathways, including nitrate, sulfate, manganase, and iron reduction, sulfur oxidation, methanogenesis, acetogenesis, and anaerobic methane oxidation (AOM) (Kotelnikova and Pedersen, 1998; Haveman et al., 1999; Hallbeck and Pedersen, 2012; Bomberg et al., 2015). In addition fungi (Pedersen, 1987) and viruses (Eydal et al., 2009) have been detected in bedrock aquifers by cultivation and DNA-based methods. Furthermore, fungal communities are suggested to be active in continental and deep subseafloor sediments (Edgcomb et al., 2010; Orsi et al., 2013), and recently, evidence of rich and active fungal communities were found in continental crystalline bedrock facture zones (Sohlberg et al., 2015).

Physical parameters influencing microbial physiology and competitiveness are temperature and pressure to which microbes need to be adapted. The increase of temperature with depth in the Fennoscandian crystalline bedrock is about 15°C per kilometer (Kukkonen, 1989). Due to the low geothermal gradient, life can potentially thrive down to several kilometers in depth in these settings. Hydrostatic pressure created by the interconnected porosity of the fracture network increases by about 100 bar per kilometer. High-pressure is not only a distinctive feature of deep subsurface environment where microbial life needs to integrate, but also makes sampling of these environments particularly challenging. While water sampling is rather straightforward, representative sampling of dissolved gases can face problems such as uncontrolled degassing and air contamination. In addition, microbial sampling has its demands depending on the method of analysis. Intact cells are needed for microscopy, RNA analysis and culturing, whereas damaged cells are applicable for DNA analysis. Contamination, the effect of pressure and sampling time also need to be controlled for.

In order to gain samples for deep biosphere studies, sampling groundwater by pumping has the advantage of yielding large volumes of water from a defined depth range, which can also be isolated by packers to abstract water from a particular fracture zone (Purkamo et al., 2013). Pressure decrease in the water line can be controlled by pumping rate, but uncontrolled gas release due to the pressure decrease can take place in the ascending water line. Deep subsurface samples at *in situ* pressure can be obtained with pressure core samplers in oceanic sediments (Reed et al., 2002; Kubo et al., 2014) and from bedrock drill holes by samplers equipped with the ability to compensate for decreasing hydrostatic pressure during sample retrieval with e.g., Positive Displacement Sampler (PDS; Regenspurg et al., 2010; Kietäväinen et al., 2013) and PAVE-samplers, pressurized water sampling system (Hatanpää et al., 2005).

In general, mines, as drained bedrock systems, have the investigational advantage that spontaneous fluid flow from drill holes made for ore exploration and evaluation is common. Thus, mines have provided an access to deep subsurface microbial ecosystems e.g., in Arctic Canada (Stotler et al., 2009; Onstott et al., 2009) and South Africa (Moser et al., 2003, 2005; Davidson et al., 2011). On the other hand, the hydrostatic pressure field around operative mines is typically disturbed, because natural and excavation related active fractures drain the surrounding bedrock.

In recent years, information on microbial communities in deep subsurface has become increasingly important as deeper parts of the bedrock are utilized in construction, for example in the case of deep storages of nuclear waste and CO_2_ as well as deep mining and production of geothermal energy. However, to date, only few sites within crystalline shields have been studied for their microbial communities below 2 km depth. Pyhäsalmi mine in central Finland provides an excellent opportunity to study microbial processes in deep subsurface crystalline rock environment. In addition to being one of the deepest mines in Europe (1400 m), near-vertical drill holes starting from the deepest levels of the mine reach the depth of more than two kilometers below the surface. Furthermore, due to the surrounding high hydrostatic pressure, the risk that some portion of the high-pressure flush water will remain in fractures and contaminate the samples is clearly smaller than in drilling from the surface. In this study microbial and geochemical samples from deep extreme subsurface mine environment from prospectively uncontaminated drill holes that produce high amounts of water were taken. The objective was to explore bacterial, archaeal, and fungal communities. The geochemical aim was to compare different geochemical sampling methods in order to find the most suitable ways of collecting fluid samples from high-pressure drill holes, as well as to reveal environmental and nutritional restrictions faced by the deep biosphere. By studying the results obtained from microbial and geochemical samples, we aimed to identify potential relationships between biological and geological factors.

## Materials and methods

### Description of the site

Pyhäsalmi Mine is situated in a volcanogenic massive sulfide (VMS) deposit, which was formed during the Paleoproterozoic era (1.93–1.92 Ga). It is located within the Fennoscandian Shield in central Finland. The stratigraphy from the lower parts of the area is composed of felsic volcanites with tuffaceous and pyroclastic rocks. The upper part of the stratigraphy is composed of mafic massive lavas, pillow lavas, pillow breccias, and pyroclastic rocks. Related tonalitic subvolcanic intrusions are also present in the area. After formation, the Pyhäsalmi deposit was subjected to multiphase deformational history where all the formations were refolded in to an upright position (Laine et al., 2015).

**Figure 1:**
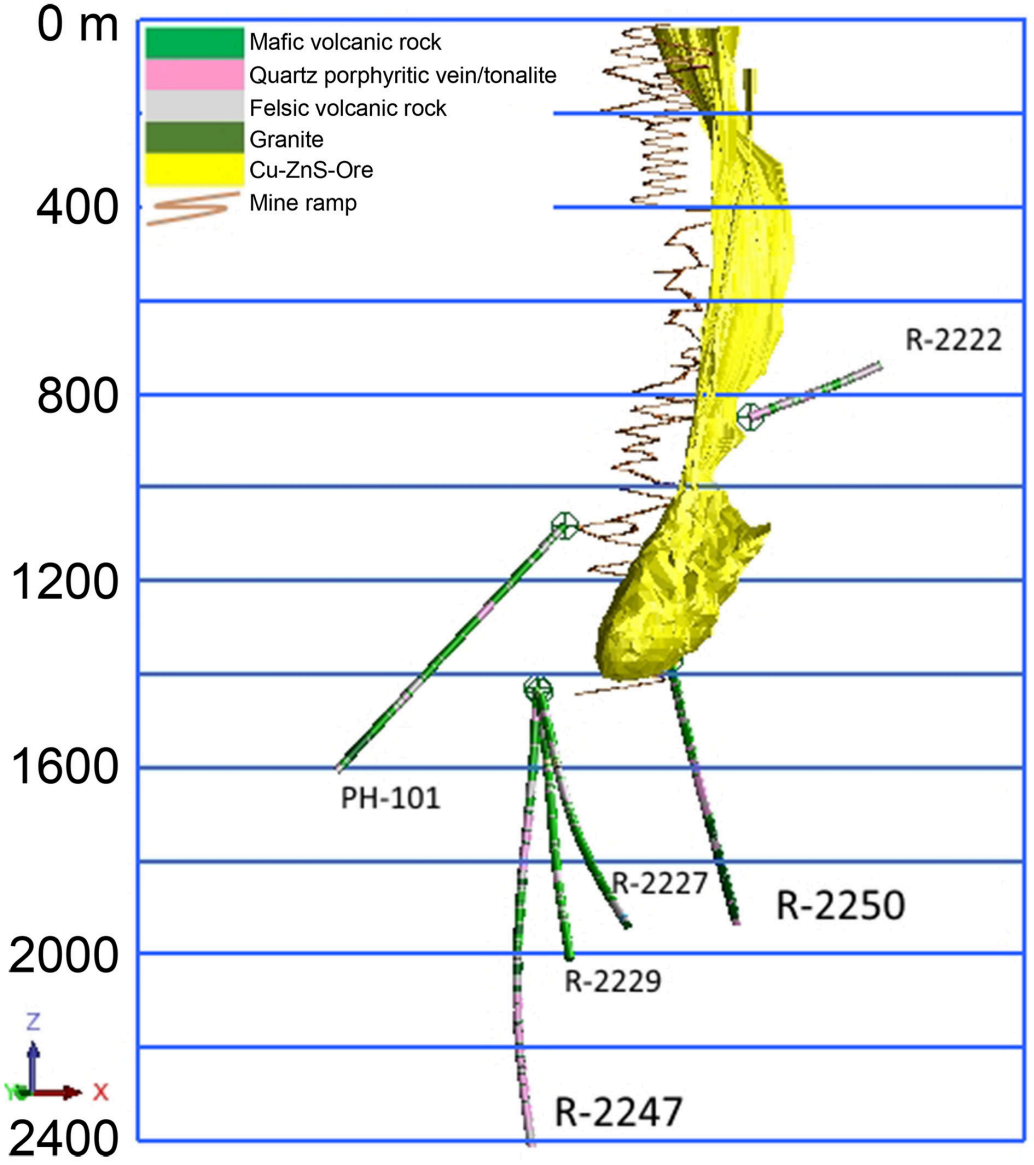
**Locations and under ground surface level depths (m) of the studied drill holes and the Pyhäsalmi ore in yellow**.

**Figure 2:**
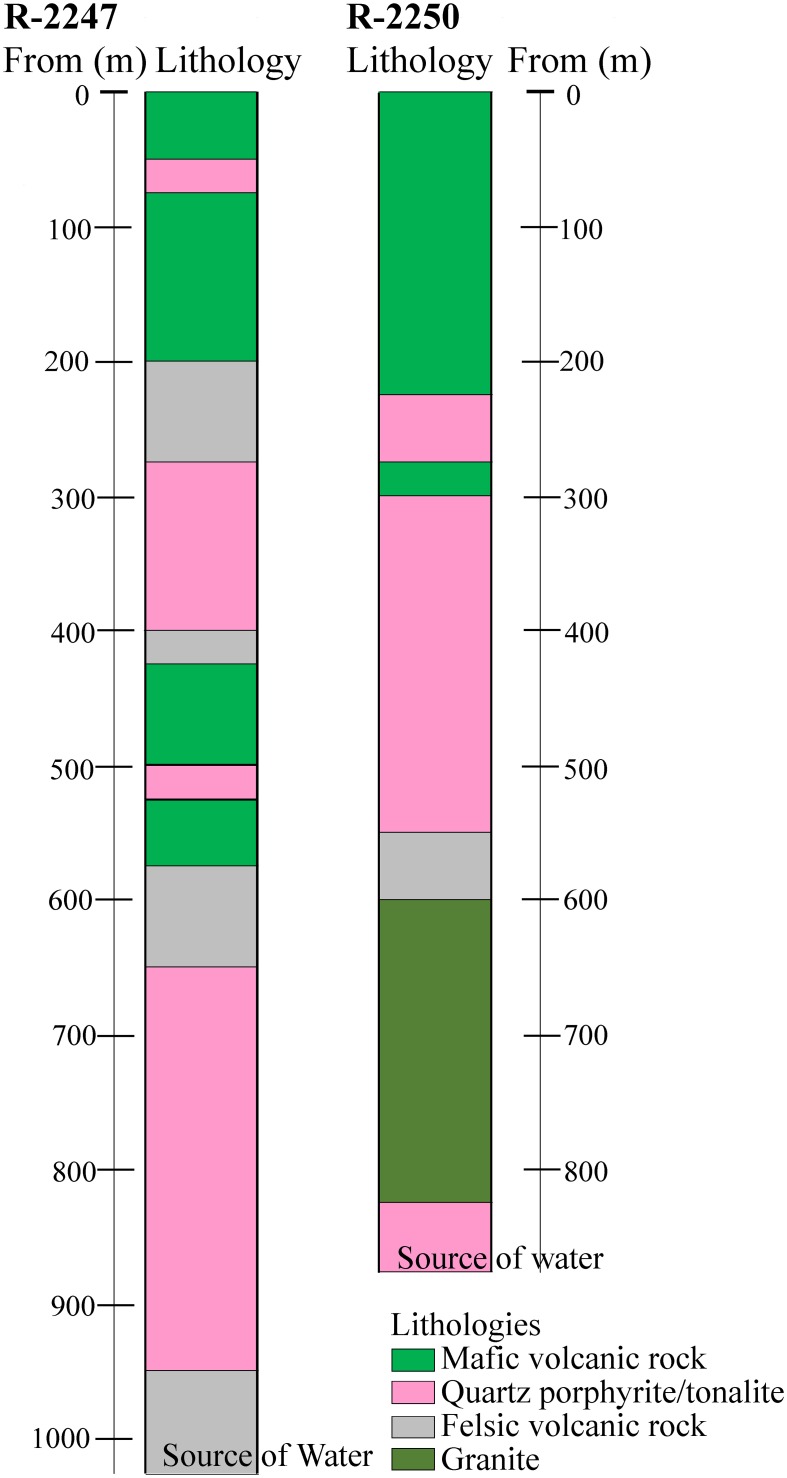
**Generalized lithologies in drill holes R-2247 and R-2250 starting from the mine levels of 1430 m and 1350 m below ground surface**.

The massive sulfide deposit (average grade: Cu 1%, Zn 2.5%, S 41%) is exploited for the production of copper, zinc and pyrite concentrates. The chalcopyrite, sphalerite, and pyrite containing ore is hosted by quartz-rich felsic volcanic rock. The main targets of the present study are the deep drill holes R-2250 and R-2247 drilled from 1350 and 1430 m levels below the surface (bsl), respectively (Figure 1). They are situated within the new deep ore body (1050–1425 m bsl) that was taken in production in 2001 and reaches a total depth of about 2000 and 2400 m bsl, respectively. The drilling method used was the common diamond drilling technique preserving a core sample of the rock, from which the rock types were determined (Figure 2). Flush water used in drilling was taken from a near-by Lake Pyhäjärvi. Diameters of the drill holes are 75.7 mm (R-2250) and 60.3 mm (R-2247). Additional drill holes situated at the level of 1430 m bsl (R-2227, R-2229), 1080 m bsl (PH-101) and 850 m bsl (R-2222) were also studied for geochemistry (Figure 1).

The drilling of both R-2250 and R-2247 was terminated because of a voluminous discharge of water from the drill hole, which exceeded the water injection pressure of the drilling machine (70 bar) and indicated that a water-conducting fracture zone was hit. The fresh drilling water was flushed out and replaced by the formation water before the drilling rods were removed and a plug was installed. The most prominent brittle tectonic feature in the area is the Oulujärvi shear zone (OSZ) (Kärki et al., 1993). It is unclear how the fracture systems that met during drilling are related to OSZ but the location (Figure 1) and lithologies (Figure 2) of the drill hole R-2250 fracture zone indicates close resemblance to it. The fracture zone perforated by the drill hole R-2247 could be evidence of an overthrust structure identified from previous seismic surveys (Laine et al., 2015). No graphite or carbonate fillings have been found in either of the fracture zones, demonstrative of a carbon-poor environment.

### Geochemical sample collection and analysis

Groundwater samples for detailed geochemical and microbiological analysis were collected from the drill holes R-2250 and R-2247 (Figure 1). Maximum pressures measured on the collars of the drill holes were 60 and 140 bar, respectively. R-2247 was sampled three times (in May and August 2013, and in June 2014) while samples from R-2250 were taken only once, in August 2013. In order to get a wider view on groundwater systematics in the mine four additional drill holes (R-2222, PH-101, R-2227, and R-2229) were studied for geochemistry. R-2222 was unplugged and was not sampled for gas composition. Samples for water stable isotopes of the drilling fluid (Pyhäjärvi lake water) were taken in February 2015.

Groundwater samples were taken directly from the free flowing fluid. Temperature, pH and electrical conductivity (EC) were measured at the site using handheld meters (WTW). Untreated groundwater samples of 500 mL each were taken for the determination of anions and alkalinity. Alkalinity was measured within the same day by titration to pH 4.5 using a digital titrator (Hach). Samples for the determination of water stable isotopes (60 mL) and cations (100 mL) were filtered (< 0.45μm, Whatman FP). In addition, cation samples were acidified with ultra pure 65% HNO_3_ (0.5 mL per 100 mL of water). Anions and cations were analyzed at Labtium Oy (Espoo, Finland) using ion chromatographic and spectrometric methods, respectively (Kietäväinen et al., 2013). Water stable isotopes were determined at the Geological Survey of Finland (Espoo, Finland) using a cavity ring-down spectrometer (Picarro) and reported as o relative to Vienna Standard Mean Ocean Water (VSMOW). Total and dissolved organic carbon were determined from unfiltered and filtered (< 0.45 μm, Whatman XP) samples (50 mL each), respectively, using a TOC analyzer at Labtium Oy. Sulfide samples were taken into 100 mL glass bottles (Winkler) and immediately fixed by adding 0.5 mL of 1 M NaOH and 0.5 mL of 1 M zinc acetate and were analyzed within 48 h at Ramboll Analytics (Vantaa, Finland) with a spectrophotometer.

Gas samples were collected in a bucket from the free flowing fluid into inverted glass bottles (Schott), which were kept under sample water, sealed with 2 cm thick black butyl rubber septa (Glasgerätebau Ochs) and closed with cut screw caps. The gas/water ratio was monitored during sampling. From the drill holes R-2247 and R-2227 gas samples were also taken by collecting the gas first into an inverted modified 1 L plastic syringe (Hamilton) and injecting it with a double-ended needle (Venoject) into evacuated glass bottles (Laborexin) sealed with blue butyl rubber septa (Bellco Glass) and aluminum open top crimp caps (Bellco Glass). A few grains of solid HgCl_2_ were added into one of the Laborexin bottles prior to evacuation in order to provide a killed control to test if biological activity affects the gas composition during storage of untreated samples. In addition, one fluid sample from R-2247, R-2229, and R-2227 each was collected into pressure tight 500 mL stainless steel cylinders (Swagelok) that were attached directly to the collar. From the cylinders, the gas was later separated into smaller 25 or 50 mL Teflon coated stainless steel cylinders (Swagelok) in a vacuum line. Gas compositions were analyzed by gas chromatography at Ramboll Analytics or Isotech Laboratories Inc. (Illinois, USA).

Based on the determinations of groundwater and gas composition, reduction oxidation pairs [C(–4)/C(4), H(0)/H(1) and S(–2)/S(6)] were modeled at *in situ* T and pH using PHREEQC software wateq4f database (USGS, 2014).

#### Microbial sample collection

In August 2013 the groundwater from the drill holes R-2247 and R-2250 was sampled as triplicate samples for DNA and RNA study. The sample line was equipped with a pressure reduction valve (Swagelok) in order to reduce the pressure to less than 2 bar. In 2014 two microbial samples were taken from the drill hole R-2247 with a different pressure reduction system. Three pressure reduction valves (Swagelok) were used and 500 mL stainless steel pressure cylinders were installed between the first and the second, and between the second and the third reduction valves to allow some time for the sample water and microbes to recover from each pressure reduction step. The recovery times were around 15–20 s. The measured pressures during the sampling in each pressure reduction step before the actual sample collection are presented in Table 1. In 2013 cells in the groundwater were collected by filtration with Sterivex filter units (Millipore Corporation, USA) and in 2014 with Steripak (Millipore) 0.22 pore size filter units connected directly to the stainless steel sampling system with a sterile ¼inch NPT to a Luer connector (Adhesive Dispensing Ltd, UK) or silicon tube, respectively. Filters were immediately frozen on dry ice after sampling and stored at −80°C until use. The sample sizes are shown in Table 1. In 2013 samples for microbial cell counts were collected in acid-washed sterile, anaerobic 100 mL glass infusion flasks through butyl rubber septa with a sterile needle. In 2014 water was collected in sterile pressure cylinders. The pressure in the cylinders was initially 90 bar but when the inlet valve was closed the pressure dropped to 50 bar in the cylinders and as the valve between the two cylinders was closed the pressure dropped to 40 bar. The residual pressure was reduced before the cell count analysis in the laboratory during a half hour in each of the six pressure reducing steps.

**Table 1:** **Collected microbial groundwater samples from two drill holes: Sampling methods, measured pressures during sampling, and sample volumes**.

**Drill hole**	**Year**	**Sampling method**	**Pressures (bar)**	**Sample volume (L)**
R-2250	2013	Filtration	60–2	0.5 (RNA), 1.0 (DNA)
		Head space bottle	60–1	0.1 (TNC)
R-2247	2013	Filtration	130–2	0.5 (RNA), 1.0 (DNA)
		Head space bottle	130–1	0.1 (TNC)
R-2247	2014	Filtration	130–90–60^a^-60-35-1	100 (DNA)
		Filtration	130–90^a^–60–35–1	155 (DNA)
		Pressure cylinder	130–90^a^–50–40	0.5 (TNC)

a *Represents a pressure drop due to gas leakage in the collar of the drill hole connection that changed during the collection. The pressure after leakage was measured every now and then and was found to be between 90 and 60 bar*. *TNC, Total number of microorganism*.

### Microbiome composition analysis

#### Total number of cells

The total number of microbial cells (TNC) was estimated by epifluorescence microscopy based on DAPI staining. The number of microbial cells was determined by fluorescent staining with 4′,6-diamidino-2-phenylindole (DAPI) (Keppner and Pratt, 1994; Itävaara et al., 2008) from the headspace bottle and pressure cylinder waters. Two 50 mL subsamples of each groundwater sample were stained with 1 μg mL^−1^ of DAPI for 20 min at room temperature in the dark under aerobic conditions and filtered on black 0.2 μm pore-size polycarbonate membrane filters (Isopore™ Membrane filters, 0.2 μm GTBP, Millipore) with a Millipore 1225 Sampling Manifold (Millipore) using low vacuum suction. The number of cells in the sample was then calculated from 30 random microscopy fields according to the magnification factor (Olympus BX60, Olympus Optical Ltd., Tokyo, Japan and 100 × magnification). The groundwater samples from 2014 (50 mL) were also stained with Live/Dead stain (Life Technologies) according to manufacturer's instructions and the portions of live and dead cells were counted.

#### DNA extraction

DNA was extracted and purified from the frozen Sterivex samples (1 L) separately from each of the triplicate samples per drill hole by aseptically breaking filter units and by cutting the filter and moving it to PowerWater DNA Isolation kit tube (MoBio Laboratiories, USA). The isolation was performed according to the manufacturer's instructions. Extracted DNA amounts were measured with a Qubit-fluorometer (Life Technologies) according to the manufacturer's instructions from 20 μL of undiluted sample.

DNA from the two Steripak filters (100 and 155 L) was separately extracted as follows. The outlet of the filter was closed with ethanol treated parafilm. 15 mL of SET-buffer (50 mM Tris-HCl, 40 mM EDTA, 0.75 M sucrose, pH 7.8) and 500 μL acid washed sterile glass beads (Sigma 150–212 μm) was added to the filter and shaken (900 r min^−1^) for 10 min at room temperature. Lysozyme (final concentration in buffer 0.8 mg mL^−1^) was added and the filter units were incubated for 30 min at 37°C. Proteinase K (final concentration in buffer 0.2 mg mL^−1^) and 1 mL of 20% sodium dodecyl sulfate was added and the incubation was continued for 90 min at 65°C. The buffer liquid was drained out from the inlet and the treatment was repeated. DNA was precipitated from the combined buffer liquids by adding 95% ethanol (78 mL) and freezing at −80°C over night. The sample was thawed and DNA was centrifuged (30 min, 11.000 g). Liquid was removed and the precipitate was washed with 70% ethanol. DNA was further purified with the PowerWater DNA Isolation kit (MoBio Laboratiories). The isolated and purified DNA was stored frozen at −80°C until use.

#### RNA extraction and reverse transcription

Total RNA from triplicate 0.5 L groundwater samples collected on the Sterivex filter units (0.22 μm pore-size) was isolated with the PowerWater RNA isolation kit (MoBio Laboratories). The filters were thawed on ice and care was taken to minimize the time of thawing. The filter units were aseptically broken and the filter membranes were removed using sterile scalpels. The intact filters were inserted into the bead tubes with flame-sterilized forceps and the RNA extraction was performed according to the manufacturer's instructions. Negative isolation controls were also included in all nucleic acid extractions. DNA contamination of the RNA extracts was checked by PCR with bacteria 16S rRNA gene specific primers U968 and U1401 (Nübel et al., 1996). If no PCR product was obtained, no DNA contamination was assumed. If a PCR product was obtained, the RNA extract was first treated with DNase (Promega) according to the manufacturer's instructions. The RNA was subsequently subjected to cDNA synthesis. Aliquots of 11.5 μL of RNA were incubated together with 250 ng random hexamers (Promega) and 0.83 mM final concentration dNTP (Finnzymes, Finland) at 65°C for 5 min and cooled on ice for 1 min. The cDNA was synthesized with the Superscript III kit (Invitrogen), by adding 4 μL 5 × First strand buffer, 40 u DTT and 200 u Superscript III to the cooled reactions. To protect the RNA template from degradation, 40 u recombinant RNase inhibitor, RNaseOut (Promega), was used. The reactions were incubated at 25°C for 5 min, 50°C for 1 h and 70°C for 15 min. Duplicate reactions per sample as well as no template controls were performed. The duplicate reactions were subsequently pooled. RT-PCR was also performed on the negative RNA extraction controls as well as negative reagent RT-PCR controls to ensure that these steps had remained uncontaminated during the process.

#### Quantitative PCR

The abundances of bacterial 16S rRNA genes were estimated by real-time quantitative PCR (qPCR). 1 μL of DNA extract was used as template from each samples as triplicate. The qPCR was performed with KAPA™ SYBR® Fast 2 × Master mix for Roche LightCycler 480 (Kapa Biosystems, USA) and triple reactions were performed for each sample. The amplifications were performed with primers P1 and P2 (Muyzer et al., 1993), which specifically target the V3 region of the bacterial 16S rRNA gene. Each reaction contained 3 pmol of each primer. The amplification was performed for 45 cycles, with denaturation (10 s at 95°C), annealing (35 s at 57°C), and extension (30 s at 72°C). The number of bacterial 16S rRNA genes was determined by comparing the amplification result (Cp) to that of a dilution series (10^1^–10^7^ copies μl^−1^) of *Esherichia coli* (ATCC 31608) 16S rRNA genes in plasmid. Inhibition of PCR was tested with comparing the amplification of standard to standard with sample liquid addition (1 μL).

#### Amplicon library preparation

The amplicon libraries for high throughput pyrosequencing were prepared by PCR from the DNA and cDNA samples from 2013. Amplicons were made as duplicates from each triplicate sample. All amplicons were produced by nested PCR as follows: the bacterial 16S rRNA genes were first amplified with primers fD1 (Weisburg et al., 1991) and U1401 (Nübel et al., 1996) and then for the V1-V3 variable regions with tagged primers 8F (Edwards et al., 1989) and P2 (Muyzer et al., 1993). Archaeal 16S rRNA gene fragments were amplified first with primers A109f (Großkopf et al., 1998) and Arch915R (Stahl and Amann, 1991) for an 806 bp long fragment of the 16S rRNA gene. Then, a second PCR with tagged ARC344f (Bano et al., 2004) and Ar774r primers (modified from Barns et al., 1994) was used to produce the tagged product for sequencing (covering the V3-V4 variable areas). For the fungal ITS fragments a 420-825 bp long fragment was first amplified with primers ITS1F and ITS4 (White et al., 1990; Gardes and Bruns, 1993). The product of this PCR was used as a template in a secondary PCR with tagged primers ITS1F and ITS2 (Buée et al., 2009) generating a ca. 400 bp product. Amplifications were also made of PCR reagent controls. The first step of PCR amplification was performed in 10 μL and second step in 50 μL reactions containing 1 × KAPA Fidelity buffer (Kapa Biosystems), 0.3 mM final concentration of dNTP, 6 pmol of each primer in 10μL reaction and 25 pmol in 50 μL reaction, 1 unit of KAPA Hifi polymeraze enzyme (Kapa Biosystems) and 1μL in 10 μL reaction, and 2 μL in 50 μL reaction of template. The PCR program for both PCR steps consisted of an initial denaturation step at 98°C for 5min, 39 cycles of 20 s at 98°C, 15 s at 53°C (for archaea and fungi at 50°C), and 30 s at 72°C. A final elongation step of 5 min was performed at 72°C. The products were cleaned with NucleoSpin PCR Clean-up kit (Macherey-Nagel) according to manufacturer's instructions. The amplicon products from the six replicates of each sample were pooled and the pyrotag libraries were sent to Beckman Coulter Genomics (USA) for pyrosequencing with a Genome Sequencer FLX 454 titanium platform according to manufacturer's protocol (454 Life Sciences/Roche Applied Biosystems).

#### Sequence processing and analysis

The bacterial and archaeal sequence reads obtained from the sequencing center were batch processed with an in-house developed pipeline (Miettinen et al., 2015). Briefly, the sequence reads were first subjected to quality control using the Mothur software version 1.31.2 (Schloss et al., 2009). Adapters, barcodes and primers were removed from the reads, and the quality of base-calls was assessed in order to remove erroneous reads from the data set. Chimeric sequence reads were identified in the data set with the USEARCH algorithm (Edgar, 2010) by *de novo* detection and through similarity searches against the 97% representative OTU set of the Greengenes reference database of 13_8 version (DeSantis et al., 2006) and were removed from the analyses. Operational Taxonomic Units (OTUs) were detected from the chimera-filtered sequence data set following the open-reference OTU-picking protocol of QIIME version 1.7.0 (Caporaso et al., 2010) against the 97% identity Greengenes 13_8 OTU sets. OTU clustering was performed with UCLUST v. 1.2.22q (Edgar, 2010), and the cluster seed sequences were selected as the representative OTU sequences. OTUs with less than two members were discarded. Taxonomy was assigned with RDP classifier v. 2.2 (Wang et al., 2007).

The raw fungal ITS sequence data were analyzed as above, except that the reference database was UNITE (Kõljalg et al., 2013), and the taxonomic assignments were made by the BLAST algorithm with a maximum *E*-value of 0.001 (Altschul et al., 1990). OTUs found in bacterial and fungal negative controls were filtered from the sample data. Alpha diversity chao1 was calculated for all microorganism types (Chao, 1984).

#### Accession number

The bacterial and archaeal 16S rDNA and 16S rRNA and fungal ITS gene region sequences have been submitted to the European Nucleotide Archive (ENA) under accession number PRJEB9160 (http://www.ebi.ac.uk/ena/data/view/PRJEB9160).

## Results

### Geochemistry

A summary of the results of geochemical characterization of groundwater in the drill holes R-2247 and R-2250 at the time of microbiological sampling is given in Table 2. A full geochemical dataset, including the other time points and drill holes, is provided in Supplementary Tables 1 and 2.

**Table 2:** **Geochemistry of groundwater in the drill holes R-2247 and R-2250 in the Pyhäsalmi mine**.

**Drill hole**		**R-2247**	**R-2247**	**R-2250**
**Sampling date**		**13.8.2013**	**11.6.2014**	**14.8.2013**
pH		8.6	8.7	8.8
EC	mS cm^−1^	103.3	102.0	34.0
Alk.	mM	0.09	0.08	0.09
TOC	mM	0.092		0.117
DOC	mM	0.066		0.085
Ca	mM	565	488	119
Fe	mM	0.0024	<0.0009	0.0006
K	mM	0.83	0.97	0.14
Mg	mM	0.14	0.13	0.04
Na	mM	324	320	112
P	mM	0.005	0.004	0.003
S	mM	1.23	1.30	1.16
Si	mM	0.14	0.10	0.18
Sr	mM	3.06	3.02	0.61
Br	mM	7.48	6.65	2.17
Cl	mM	1272	1097	285
SO_4_	mM	1.78	1.58	1.23
Sulfide	mM	0.0023	0.0020	0.057
TDS	g L^−1^	76.2	66.9	17.7
Gas_*tot*_	mL L^−1^	56	119	60
Ar	mL L^−1^	0.58	1.06	0.40
O_2_	mL L^−1^	0.31	0.40	0.19
N_2_	mL L^−1^	39	73	26
H_2_	mL L^−1^	0.10	9.0	0.74
He	mL L^−1^	14	30	19
CH_4_	mL L^−1^	1.6	3.9	14
C_2_H_6_	mL L^−1^	0.24	0.46	0.08
C_3_H_8_	mL L^−1^	0.007	0.014	0.002
Iso-butane	mL L^−1^		0.0002	
N-butane	mL L^−1^		0.0012	
N-pentane	mL L^−1^		0.00012	

*EC, electrical conductivity; Alk, alkalinity; TOC, total organic carbon; DOC, dissolved organic carbon; TDS, total dissolved solids*.

**Figure 3:**
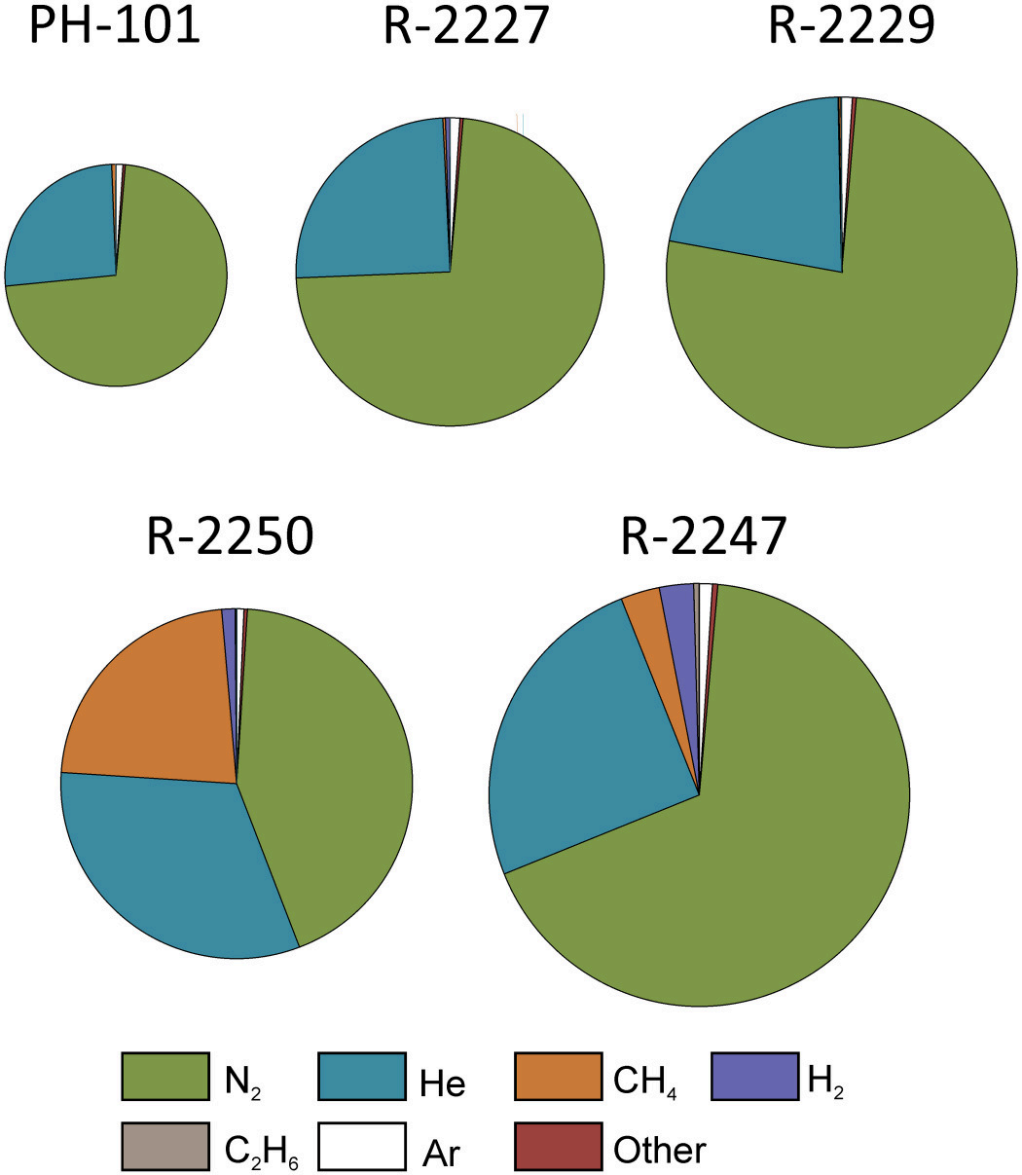
**Relative compositions of dissolved gases in the drill holes sampled in the Pyhäsalmi mine**. Areas of the charts are proportional to the average gas volumes measured in each of the drill holes, with the largest chart (R-2247) corresponding to a gas volume of 90 mL per 1 L of groundwater. Other gases include O_2_, CO_2_, propane, butane, and pentane (Supplementary Table 2).

*In situ* temperature at the sampling sites and of the groundwater was approximately 23°C at 1430 and 1350 m bsl. Groundwater from R-2247 was saline [total dissolved solids (TDS) up to 76 g L^−1^] and of Ca-Na-Cl type. Water from the drill hole R-2250 was approximately 4 times more dilute, less Ca-dominated and more sulfide rich compared to R-2247. Water from the both drill holes contained 1–2 mM of sulfate. Nitrate was below detection, i.e., <0.16 and <3.2 mM in R-2247 and R-2250, respectively. Based on alkalinity and TOC measurements carbon contents were found to be low. The amount of DOC was 70–80% of the total organic carbon. Dissolved gas phase in both R-2250 and R-2247 was comprised mostly of nitrogen and helium (Figure 3). R-2250 also contained a significant amount of methane. CO_2_ was detected only in two samples, which were either fixed with HgCl_2_ (PYS-1B, R-2247) or collected using a pressure cylinder (PYH-2A, R-2229; Supplementary Table 2). Small amounts of oxygen were present in all other samples but the one sample pressurized from R-2247. However, based on C(–4)/C(4), H(0)/H(1), and S(–2)/S(6) redox pairs, *Eh*-values are between –0.3 and –0.4 V in both R-2247 and R-2250 indicating clearly reductive conditions. CO was not detected but small amounts of C_2+_ hydrocarbons up to pentane (C_5_) were found. The amount of gas in the drill hole R-2247 in 2014 was approximately doubled compared to the same sample in August 2013. The most notable change was the increase in H_2_, while relative amounts of other gases stayed relatively constant. Furthermore, He and H_2_ were more enriched in the pressurized samples compared to other samples taken at the same time.

**Figure 4:**
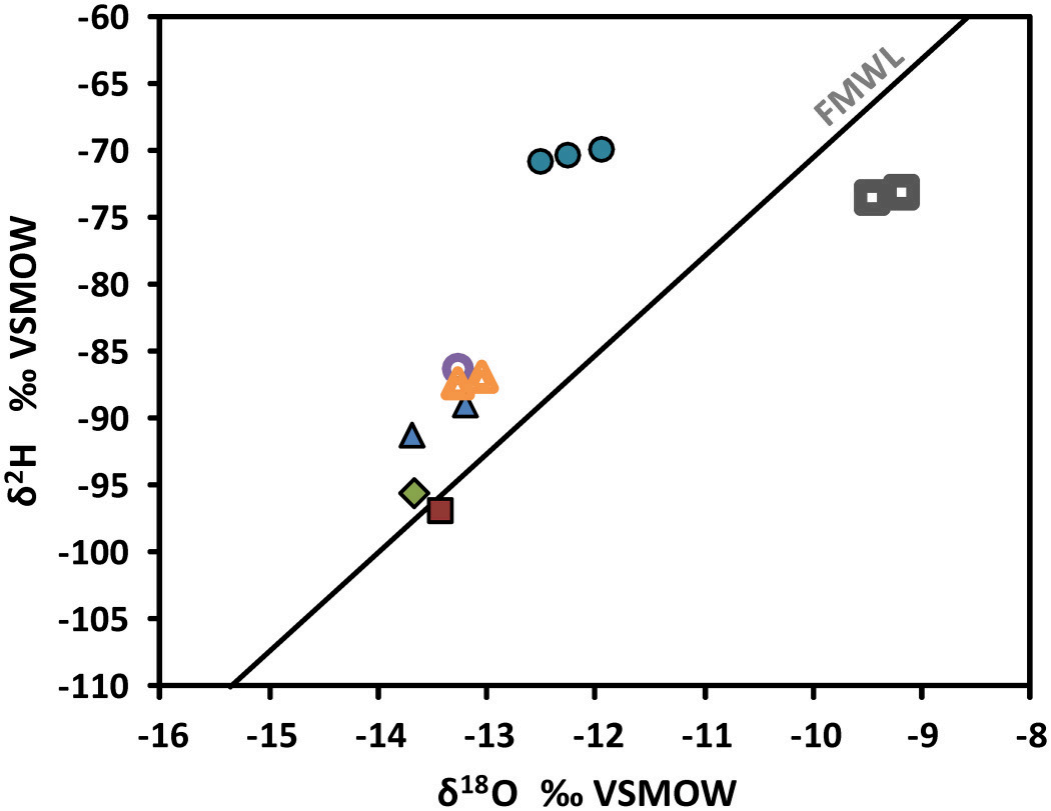
**Stable isotope compositions of groundwaters in the Pyhäsalmi mine**. Values are given in o relative to Vienna Standard Mean Ocean Water (VSMOW). FMWL, Finnish Meteoric Water Line (Kortelainen, 2007) depicts the average composition of precipitation in Finland. Most of the samples plot left from the FMWL. This is typical for deep saline groundwaters within crystalline rocks, which have been modified by water-rock interaction (e.g., Frape et al., 2003). Circles = R-2247, open circle = R-2250, triangles = R-2227, open triangles = R-2229, diamond = PH-101, square = R-2222, open squares = drilling water from Lake Pyhäjärvi.

Within groundwater samples δ^2^H and δ^18^O vary from −96.8 to −69.8o VSMOW and −13.70 to −11.96o VSMOW, respectively (Supplementary Table 1). Isotopic composition of the most saline water (R-2247) plots clearly left from the Finnish Meteoric Water Line (FMWL, Kortelainen 2007) while groundwaters sampled from the shallower levels of –1080 m (PH-101) and –850 m (R-2222) feature compositions typical for meteoric, fresh groundwater (Figure 4). Both compositions are clearly distinct from the lake water used for drilling with δ^2^H of −73.3o VSMOW and of δ^18^O of −9.33o VSMOW.

### Microbiology

Microbial cells could not be detected in the water samples from 2013 by microscopy. Only some blurred stains were visible that could not be identified as cells. In 2014 the number of microbial cells detected in the groundwater by microscopy was 1.4 × 10^4^ cells mL^−1^ at R-2247 (drill hole bottom at ~2400 m bsl) from the pressure cylinder groundwater stained with DAPI. The amount of live cells determined with Live/Dead staining was 1.2 × 10^4^ cells mL^−1^ and the injured cell count was 1.5 × 10^2^ cells mL^−1^.

The amount of isolated DNA from both of the drill holes (2013; 1 L samples) was less than 0.05 ng. In 2014 the total isolated DNA amount from 100 and 155 L groundwater was 1.1 and 1.3 ng, respectively. Even though more DNA was obtained from the samples from 2014 compared to those from 2013, no PCR product was obtained from the 16S rRNA gene qPCR in either year. However, no PCR inhibition was seen. As 16S rRNA qPCR was not successful no other qPCRs were performed for marker genes. Amplicon sequencing analysis targeting the bacterial and archaeal 16S ribosomal genes and fungal internal transcribed spacer (ITS) gene region were done with the nested PCR products of the year 2013 samples. As no products were obtained from the year 2014 samples with 16S rRNA gene qPCR, the samples were considered not to represent the original drill hole diversity any better than the year 2013 samples and no high-throughput amplicon sequencing analysis was performed on these samples.

**Figure 5:**
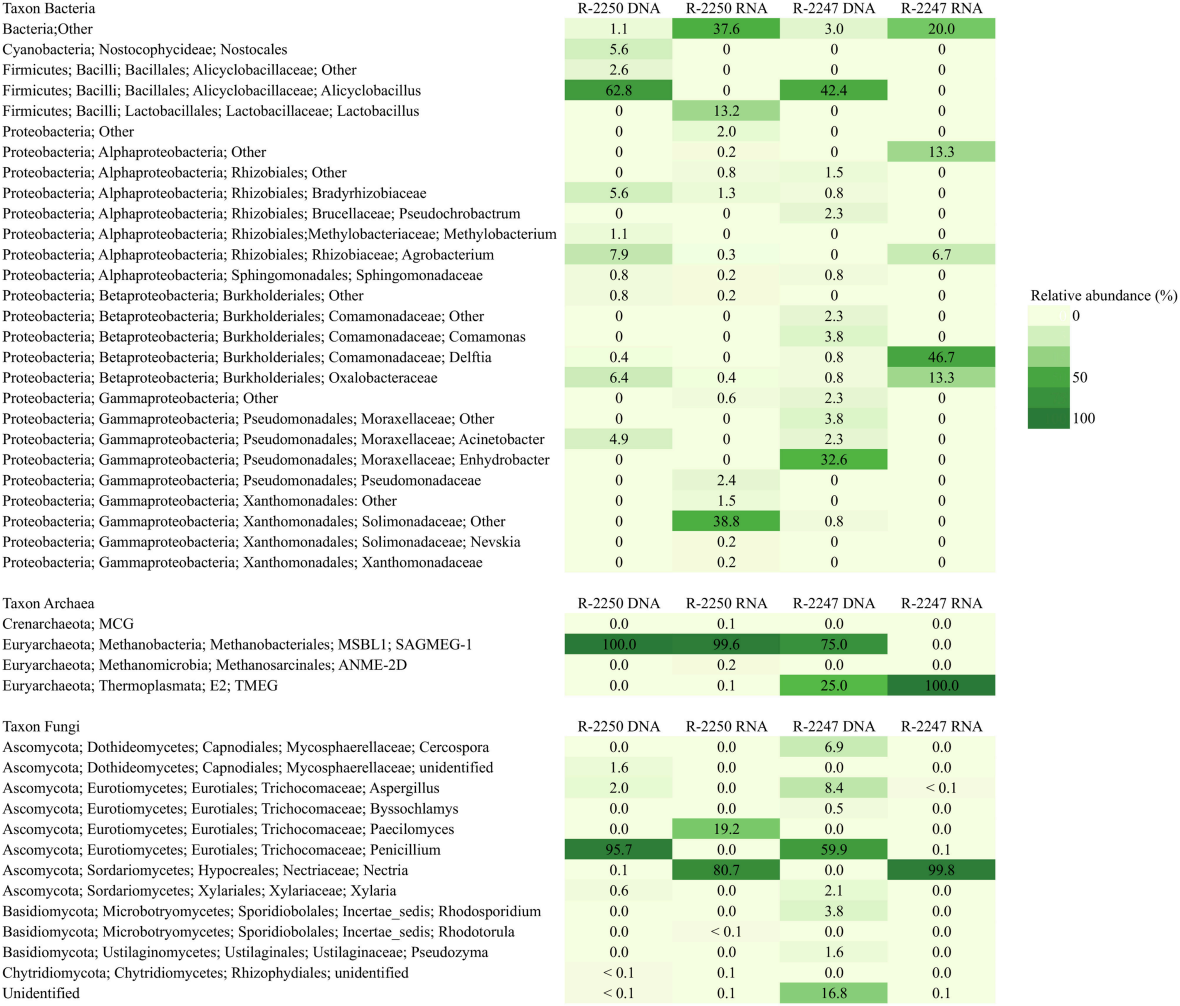
**Bacterial, archaeal, and fungal taxonomy in deep groundwaters of the Pyhäsalmi mine in drill holes R-2247 and R-2250**. Taxonomic classification of the bacterial, archaeal, and fungal sequence reads obtained by high throughput sequencing of the total (DNA) and potentially active (RNA) communities presented at the genus level.

Bacterial and archaeal 16S rRNA gene sequences were obtained from all samples. Most of the sequences were obtained from the shallower drill hole R-2250, consisting of around 3000 archaeal sequences from both DNA and RNA fractions but less than a thousand sequences were obtained from each bacterial amplicon library (Supplementary Table 3). From the deeper drill hole R-2247 the sequence amounts from each sample (< 138 sequences) serve only as an exemplary of the drill hole prokaryotic diversity. Fungal sequences dominated in both drill hole samples (over 11,700 sequences from each sample type). Rarefaction curves from all samples with more than 300 sequences are presented in Supplementary Image 1. Different operational taxonomic unit (OTU) counts were lowest in the case of archaea (<7 OTUs) and the highest number of OTUs was found in deeper R-2247 fungal DNA fraction (77 OTUs). The bacterial, archaeal, and fungal community compositions are shown in Figure 5.

Bacterial diversity was low and the highest number of detected OTUs was only 45 OTUs found from R-2250 with 946 sequences. The sequences found in all samples grouped into three phyla: Proteobacteria, Firmicutes, and Cyanobacteria (Figure 5). All samples included also unidentified bacterial sequences especially in the RNA fraction describing a potentially active community (20–38% of the sequences). In both drill holes the DNA fraction describing the total community was dominated by sequences affiliating with the phyla Firmicutes and Proteobacteria. The majority of the bacterial 16S rRNA sequences (63 and 42%) were classified to genus *Alicyclobacillus* of the phylum Firmicutes in both R-2250 and R-2247, respectively. The bacterial diversity detected from the RNA fraction was higher than that of the DNA fraction in R-2250. The sequence reads belonged especially to Gammaproteobacteria (44%) and Firmicutes (13%) lineages. The Solimonadaceae (39%) was the most abundant separate family.

The archaeal diversity was very low. The number of sequences (5) found from R-2247 does not describe any real diversity in this sample. In R-2250 sample the sequencing depth seemed to be sufficient (over 3000 sequences in each sample) as the rarefaction curve stabilized and the chao1 estimate was the same as the actual number of identified OTUs (Supplementary Image 1 and Supplementary Table 3). However, the archaeal diversity was very low as only 4 and 6 OTUs were found in DNA and RNA fractions, respectively. These OTUs belonged to three euryarchaeotal orders: Methanobacteriales, Methanosarcinales, and Thermoplasmata and one OTU to crenarchaeotal MCG class. The majority of the archaeal sequences belonged to the order Methanobacteriales.

Fungal diversity detected from the DNA fraction was highest in R-2247 with 77 OTUs identified. In R-2250 28 OTUs were detected in the DNA fraction and 13 OTUs in the RNA fraction. The sequencing efficiency was appropriate for the fungal communities as the number of detected OTUs was close to the chao1 estimated OTU numbers (Supplementary Table 3). Ascomycota was the dominating fungal phylum detected in both of the drill holes (Figure 5). The dominating ascomycotal class was Eurotiomycetes (67 and 98%) in DNA fraction in both of the drill holes. These OTUs related most closely to genera *Penicillium* and *Aspergillus*. Sequences similar to the fungal class Dothideomycetes (7%) were detected in R-2247 in the total fungal community. Basidiomycota constituted the minor fungal phylum detected in the R-2247 DNA fraction (5%). Basidiomycota sequences belonged to Microbotryomycetes and more specifically *Rhodosporidium*-like yeasts. 17% of the sequences in the DNA fraction of the R-2250 were identified to represent other fungal phylotypes. However, their exact phylogenetic affiliation remains elusive. In the active fungal community of both of the drill holes most of the fungal sequences were similar to ascomycotal class Sordariomycetes and more closely to genus *Nectria*. In R-2250 in the RNA fraction also fungal sequences belonging to ascomycotal class Eurotiomycetes and to genus *Paecilomyces* were found.

## Discussion

### Geochemical aspects on representativeness and limits of the deep biosphere

Deep fluids from the Pyhäsalmi mine have several features in common with other deep Precambrian continental subsurface environments (e.g., Nurmi et al., 1988; Frape et al., 2003): they are saline, Ca-dominated and have water stable isotope compositions left from the meteoric water line. Even though the dissolved gas phase is dominated by nitrogen, the gas composition clearly differs from the atmosphere. These features may indicate long-term water-rock interactions. Estimated residence times of saline fluids within the Fennoscandian Shield vary between 4 and 60 Ma (Kietäväinen et al., 2014; Trinchero et al., 2014). High concentrations of helium likely indicate similar residence times of the saline groundwaters also analyzed in our study. Even though we cannot exclude other sources due to a lack of information on the isotopic composition of helium, it is likely that this helium originates from long-term accumulation due to radioactive decay of U and Th in the crust (e.g., Ballentine et al., 2002). No mantle helium was detected from the 2.5 km deep Outokumpu Deep Drill Hole located within the Precambrian bedrock some 160 km SE from the Pyhäsalmi mine in a similar tectonic setting (Kietäväinen et al., 2014).

Small amounts of oxygen detected in the gas samples likely originate from atmospheric contamination during sampling or analysis. This is because no O_2_ could be found in the pressure cylinder sample collected from R-2247. This sampling technique does not include any injection of sample into sample container or analysis line, which clearly decreases the risk of contamination. However, not all pressure cylinder samples were O_2_ free. Further evidence of strictly anaerobic conditions is brought by reduction oxidation pairs of carbon, hydrogen and sulfur, which give negative values down to −0.4 V, indicative of clearly reductive conditions. The hydrogen pair gives the most negative values, which could be related to the mobility of H_2_ in the system and thus redox disequilibrium within the samples.

It was observed that gases with the lowest solubility, i.e., H_2_ and He, were preferably found in the pressurized samples. This is likely an indication of escape of these gases during sampling from free flowing fluid. However, it may also be due to partial degassing and preferential enrichment of less soluble gases during gas separation from the pressure cylinders. Variability in the concentration of H_2_ between different samplings can also be explained in terms of solubility as H_2_ is easily enriched if there is a gas pulse. Such a gas pulse might be caused by a pressure drop within the formation. In a mine environment this may include opening of fractures due to blasting and drilling operations. These operations might also trigger chemical reactions between freshly crushed rock and water, releasing H_2_ (Kita et al., 1982; Hirose et al., 2011). Episodic release of H_2_ from fracture networks has been proposed to provide energy for deep microbial communities within crystalline shields (Sherwood Lollar et al., 2007). H_2_ can accumulate into sealed pockets within crystalline bedrock as a result of radiolysis of water (Lin et al., 2005a,b) or through reactions including oxidation of Fe(III)hydroxides (e.g., Mayhew et al., 2013). Due to the co-existence of H_2_ with high amounts of most likely radiogenic helium, the radiolytic origin seems more probable for H_2_ in the Pyhäsalmi mine.

**Figure 6:**
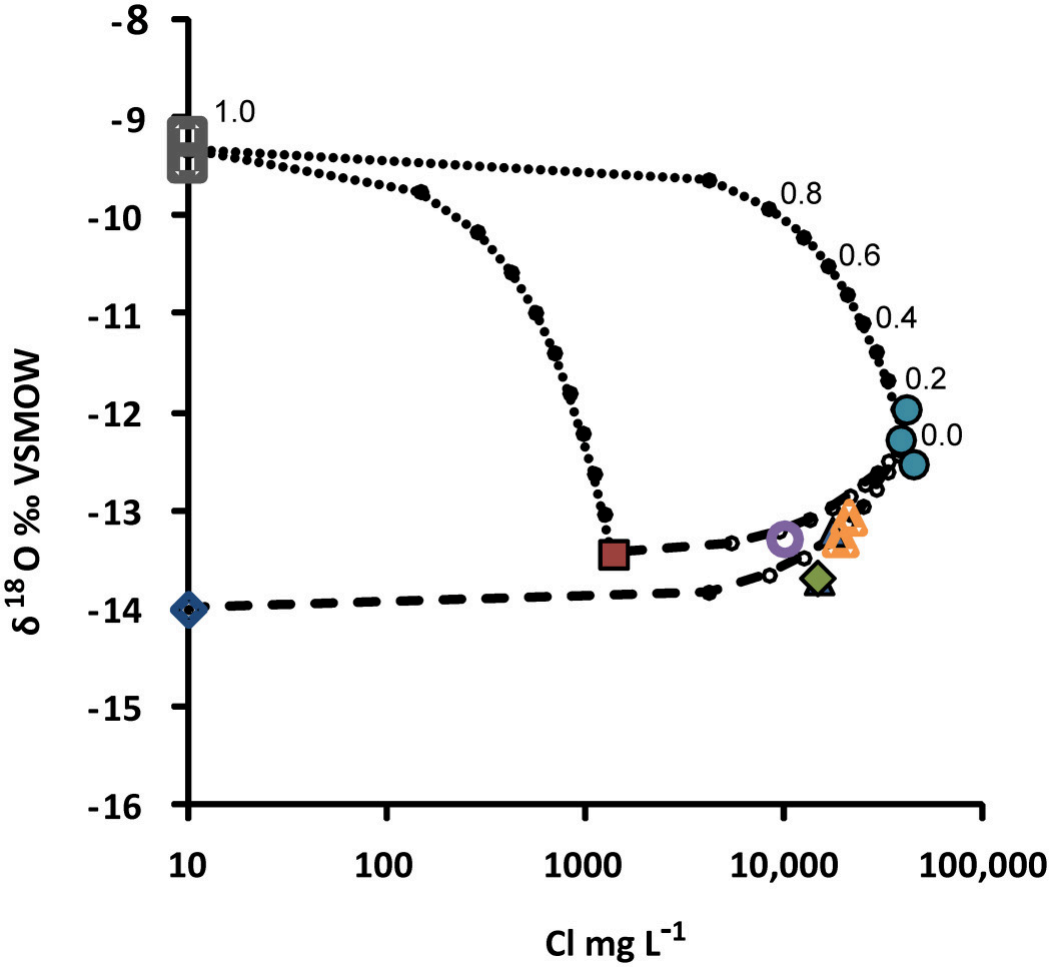
**Mixing model for groundwaters collected from the Pyhäsalmi mine**. The model is based on oxygen isotopic composition of water (δ^18^O o Vienna Standard Mean Ocean Water, VSMOW) and salinity (represented by the concentration of Cl). End-members used are (1) drilling water (open squares), (2) deep saline formation water (R-2247, circles), (3) shallow groundwater (R-2222, square), and (4) approximated composition of local precipitation/fresh groundwater (open diamond). Due to the semi-logarithmic scale, simple two-component mixing appears as a curve in this plot. Mixing of drilling water with the end-members (2) and (3) is illustrated by the dotted lines, and mixing with the end-member (2) with (3) and (4) is shown by the dashed lines. Proportions of the end-members are indicated by small circles, as exemplified for the mixing between (1) and (2). Composition of groundwater from the drill hole R-2250 (open circle) can be explained by mixing of the end-member (2) with either (3) or (4), with the proportion of saline formation water around 0.2 or 0.3 i.e., 20 or 30%, respectively, without significant mixing with drilling water. Symbols are as in Figure 4.

Despite the carbon poor conditions indicated by the lack of carbon-rich fracture fillings and only minor amounts of dissolved carbon detected in the formation waters, some methane was found especially in the drill hole R-2250 (Table 2). There a surface driven, more carbon-rich groundwater may mix with deep sourced H_2_-rich formation water (Figure 6) producing methane either through microbial metabolism or abiotically. Microbial involvement may be substantiated by the presence of CO_2_ in the HgCl_2_ treated sample, but not in the comparative samples. However, more data would be needed to confirm this relationship.

Compared to natural variability, no dramatic differences were observed between the different gas sampling methods. Thus, for a simple characterization of gas composition the most convenient way to collect samples is from a free flowing fluid. However, changes may appear in the amounts of the least soluble gases as discussed above. Drawbacks of the pressurized method include the laborious separation of gas from the sample cylinder and the relatively small sample volume obtained. In addition, quantitative gas separation still needs to be confirmed. Nevertheless, dissolved gas phase forms the most interesting part of the fluid geochemistry in terms of availability of energetic compounds in deep subsurface environments.

### Microbial diversity

The bacterial 16S rRNA gene diversity was low and only describes the most abundant species or the cells most resistant to the decompression. Despite the low sequence counts the diversities in the total community of the drill holes R-2247 and R-2250 were quite similar. The dominating OTU was a sequence similar to the Firmicutes genus *Alicyclobacillus*. Representatives of this genus inhabit mostly acidic geothermal environments (Imperio et al., 2008) and a few strains of *Alicyclobacillus* are known to reduce nitrate to nitrite and some strains also reduce Fe^3+^ (Da Costa and Rainey, 2009). *Alicyclobacillus* are spore-forming, which may explain its abundant presence due to better decompression tolerance. In addition, alphaproteobacterial orders Rhizobiales and Sphingomonadales, betaproteobacterial order Burkholderiales, and gammaproteobacterial order Pseudomonadales sequences were frequently found in the DNA fractions of both samples. These lineages are often found in deep subsurface terrestrial aquifers (Moser et al., 2003; Itävaara et al., 2011; Nyyssönen et al., 2014). A small portion of the sequences (6%) in R-2250 was similar to Cyanobacteria and was further grouped to order Nostocales. Low numbers of sequences belonging to the Cyanobacteria have previously been reported from deep extreme environments (Kormas et al., 2003; Bomberg and Itävaara, 2013), however their presence as photosynthetic bacteria in the deep dark subsurface seems unclear and their role is not known.

The diversity of the potentially active community was higher than that of the community detected from the DNA fraction in the shallower drill hole R-2250 (Figure 5). There were especially sequences similar to Gammaproteobacteria (44%) and Firmicutes (13%), the gammaproteobacterial Solimonadaceae (39%) being the most abundant separate lineage. Members of the Solimonadaceae are usually associated with soil and freshwater and described for their ability to decompose chemical pollutants (Zhou et al., 2014). The RNA fraction of the sample from R-2247 yielded only few sequences of which most grouped with betaproteobacterial *Delftia* genus. Similar sequences have been found from river estuary sediment in southern China (GenBank accession EU880508.1) and saline hydrothermal water in a Mexican mine (Ragon et al., 2013).

Archaeal sequences obtained in the deeper drill hole R-2247 identify only a few species likely present in this environment and give a hint that there might be more archaea to be found. In R-2250 the sequencing was more successful but again only a portion of the diversity was detected as the initial DNA and RNA amounts were small. The majority of the archaeal sequences belonged to the Euryarchaeota while a low number of crenarchaeotal sequences were detected only in R-2250 (Figure 5). Almost all sequences from R-2250 DNA and RNA fractions and also in R-2247 DNA fraction belonged to the Methanobacteriales order, which are generally hydrogenotrophic and use H_2_ to reduce CO_2_ to CH_4_ (Bonin and Boone, 2006). These sequences were further affiliated within the SAGMEG1 (South African Cold Mine Euryarchaeal Group 1) group that was first detected in deep South African gold mines (Takai et al., 2001). The first of the two additional rare euryarchaeal groups found from R-2250 RNA fraction was similar to the Methanomicrobia class and was further grouped with the anaerobic methane oxidizing ANME-2D family. The second rare sequence type was similar to the Thermoplasmata and belonged to the E2 order, which is known to associate with anoxic environments and includes methanogens (Yashiro et al., 2011).

More fungal than bacterial and archaeal OTUs were detected in the deeper R-2247 drill hole (82 and 21 OTUs in DNA and RNA fractions, respectively). Moreover, the fungal diversity was higher in R-2247 than in R-2250 contrary to the bacterial and archaeal diversities. Similar or higher fungal diversity (7–163 OTUs) have been detected in the Fennoscandian deep subsurface groundwater in several drill holes using the same 454 pyrosequencing method for fungal ITS regions as used here (Sohlberg et al., 2015), and up to 43 fungal OTUs in deep-sea environments have been detected by sequencing clone libraries constructed with three different primer sets of the fungal ITS gene region (Nagano et al., 2010). The dominating fungal class in the total community of both of the drill holes was Eurotiomycetes and these sequences were most closely related to *Penicillium* and *Aspergillus*. These fungi are known to be ubiquitous and cosmopolitan and function in virtually all ecosystems. They have been detected in deep-sea environments where they were found to be adapted to the aquatic environment (Damare and Raghukumar, 2008) and to tolerate high salt concentrations and anaerobic conditions (Raghukumar, 2012) that also prevail in the Pyhäsalmi mine bedrock. Another abundant sequence type found from the total community of the drill hole R-2247 was affiliated with the class Dothideomycetes and was closely related to the genus *Cercospora*. Species within the *Cercospora* act as parasites of plants and are globally distributed and spores of *Cercospora* are easily aerosolized (Kellogg and Griffin, 2006). Their role in deep terrestrial groundwater is unknown. The total community in R-2247 also included fungal sequences similar to the Basidiomycota phylum, which is closely related to the genera *Rhodosporidium* and *Pseudozyma. Rhodosporidium* is the sexual state of several species of *Rhodotorula*. The genus *Rhodotorula* is thought to be of polyphyletic origin and is phylogenetically a mixed grouping with the genus *Rhodosporidium* (Nagahama et al., 2001). These yeasts have been previously detected with cultivation-based methods from groundwaters within Fennoscandian crystalline rocks (Ekendahl et al., 2003) and from deep-sea environments (Nagahama et al., 2006). Both *Rhodosporium*- and *Pseudozyma*-species have been detected in deep-sea environments (Nagahama et al., 2001) and brackish and sea water environments (Fell et al., 1988). Interestingly, *Rhodosporium*—species have been able to grow at as high as 400 bar pressure (Lorenz and Molitoris, 1997).

In the active community the dominant fungi belonged to the class Sordariomycetes (81–99%) and more closely to the genus *Nectria* in both of the drill holes. The Nectricea includes facultative anaerobic microscopic fungal genera able to use nitrate or nitrite as a final electron acceptor as an alternative to oxygen (Kurakov et al., 2008). In terrestrial environments they act as parasites of plants and are saprophytes. Their origin and role in the deep terrestrial environment is unknown although *Nectria* -species have been isolated from deep-sea sediments (Singh et al., 2012). The active community in the shallower drill hole also has a smaller amount (19%) of sequences grouping with the class Eurotiomycetes and further classified to genus *Paecilomyces*. *Paecilomycetes*—species have been isolated from marine sediments. They have been found to be capable of growing in anoxic conditions and to perform denitrification (Cathrine and Raghukumar, 2009). Overall, the dominant fungal taxa found both in the DNA and RNA fractions were also detected abundantly in a study from Fennoscandian Shield crystalline bedrock in Olkiluoto, Finland (Sohlberg et al., 2015). This indicates that these fungal classes could be characteristic to the deep terrestrial biosphere.

This study performed in the Fennoscandian Shield crystalline bedrock, with groundwater samples from 2.4 km below the ground surface level with high salinity (TDS up to 76 g L^−1^) and especially high calcium and magnesium concentrations (22 and 0.4 g L^−1^, respectively), found the bacterial taxa *Alicyclobacillus* genus and Solimonadaceae that are not found as dominant bacterial groups in the Fennoscandian Shield bedrock studies earlier. The fungal taxa identified here provide further evidence for the fungal core microbiome detected recently from the Fennoscandian Shield bedrock (Sohlberg et al., 2015). In addition, the fungal genus *Cercospora* was detected. To the best of our knowledge, this genus has not been found in either deep subsurface bedrock or sediment environments before.

### Microbial sampling in high-pressure environment

The microbiological results of the first sampling session were surprising as no recognizable microorganisms were microscopically detected. In addition, very low DNA and RNA amounts were obtained from the groundwater filtration samples and microbial communities could only be detected with a nested PCR approach. It is possible that the abrupt pressure drop of 58 or 128 bar during sampling may have ruptured at least a part of the microbial cells and therefore the nucleic acids could escape through the filters. Hemmingsen and Hemmingsen (1980) have shown that fast (1–2 s) decompression from gas saturation pressures (25–50 bar) ruptured cell envelopes and decreased the counts of viable cells of three different species of Gram negative gas vesicle containing bacteria. Pressure drops between 50 and 100 bar ruptured the majority of these cells, however cells that did not have gas vesicles were not affected by decompression from gas saturation pressures of up to 300 bar (Hemmingsen and Hemmingsen, 1980). However, the cell envelope can be ruptured regardless of whether a membrane-enclosed vacuole is present as was seen in the case of a deep-sea methanogen *Methanococcus jannaschii* that ruptured upon rapid decompression (1 s) from 260 bar of hyperbaric pressure to atmospheric pressure (Park and Clark, 2002). To avoid this possible reason for the low nucleic acid yield we added two pressure-decreasing steps for the second sampling session. In practice altogether four pressure-reduction steps occurred as the drill hole's first joint started to leak during the microbiological sampling and gases were partly escaping which caused a pressure drop estimated to be as high as 40–70 bar. We also included some time difference between the pressure reductions by placing cylinders between the pressure reduction points.

In addition to having a better control of the pressure, we wanted to maximize the amount of harvested microorganisms from groundwater with extremely low microbial counts by collecting larger sample volumes. Thus, we used Steripak filter units that are designed for pressure driven tissue media filtration but are substantially faster and have bigger filtration capacity than that of the small Sterivex filters. However, the filters cannot be cut out from the Steripak filter unit and moved to commercial extraction kits. Instead, the microbial cells need to be released and lysed to the liquid phase inside the intact filter unit. To ensure that we would get at least a confirmation that there really were microorganisms present in the Pyhäsalmi mine groundwater, we took a small water sample without the harsh pressure decrease procedure into a pressure cylinder and lowered the residual pressure with small steps in the laboratory. The microbial cell count from this pressure cylinder was 1.4× 10^4^ cells mL^−1^ (DAPI) with 1.2 × 10^4^ live cells mL^−1^ (Live/Dead) from the R-2247 drill hole. The injured cells were a minority (1.5 × 10^2^ cells mL^−1^). This cell count is consistent with the average cell counts from this depth of groundwater (McMahon and Parnell, 2013) and is 10-fold higher than that detected from 2.3 km depth from the Outokumpu Deep Drill Hole (Purkamo et al., 2013; Nyyssönen et al., 2014). This also confirms that microorganisms are present in the deepest groundwater of the Pyhäsalmi mine.

Another reason for the low DNA and RNA amounts isolated might be the filter breakage during sampling. However, this seems unlikely as the maximum inlet pressure for both used filter types is over 3 bar, according to the manufacturer, and during sampling the pressures were mainly 1 bar and at most 2 bar. A more probable cause for the low DNA and RNA extraction amount could be inhibitory substances within the filtered groundwater, as the water was very saline with a calcium concentration over 20 g L^−1^. Both microbial cells and nucleic acids tend to have high adsorption on soil components (Frostegård et al., 1999). This may be especially relevant if part of the cells were damaged during collection and nucleic acids became susceptible to environmental conditions. The adsorption of nucleic acids to surface-reactive soil fractions such as mineral matrixes can result in low extraction yields (Direito et al., 2011). The filters collecting samples overnight were turned from white to gray and almost black throughout indicating particle accumulation and/or chemical reactions occurring on the filter surface. Especially in clay silicates (Direito et al., 2011) and iron rich clays, DNA and RNA yields can be minimal or absent, primarily due to nucleic acid adsorption (Hurt et al., 2014). The polyanionic property of the phosphate groups of the nucleic acids support a large number of binding sites for divalent or multivalent cations that further bind to negatively charged mineral particles (Hurt et al., 2014). The effect of mono- or divalent cations has also been shown to be dependent on the species of cation species, with a greater adsorption of DNA in the order of Ca^2+^, Mg^2+^ and Na^+^ (Paget et al., 1992; Nguyen and Elimelech, 2007). The Pyhäsalmi mine groundwater contained a high concentration of Na^+^ and especially Ca^2+^. It is probable that at least part of the DNA was bound to mineral particles due to presence of high Ca^2+^ concentration. To prevent DNA adsorption supported by cations, chelating agents removing metal ions from aqueous solutions, are often incorporated into the lysis buffer. The extraction method used for Steripak filters included some chelating EDTA in the lysis buffer but more attention could have been paid to desorption of DNA from the solid particles. The commercial isolation kit used was functioning in terms of purification as no PCR inhibition was found. However, efficient purification is again a source of decreased DNA yield.

There were probably several reasons for the very low nucleic acid gain as described above. However, the most probable reason for this was the strong pressure drop causing rupture of at least part of the cells and nucleic acid drift through the sampling filters. This is supported by the fact that no whole cells were seen by microscopy in decompressed water sample compared to clear cells seen in water collected into pressure cylinder without harsh decompression. In addition higher sequence counts were obtained from the drill hole R-2250 than from R-2247 as the pressure drop during sampling in R-2250 was half of that compared to the deeper R-2247.

### Microbial contamination of drill holes

In the Pyhäsalmi mine the possible microbial contamination of the drill hole waters has to be considered as the drill holes were plugged and not continuously flowing after drilling. Contamination due to drilling water is relevant especially in R-2250 as it was newly drilled. However, the high hydrostatic pressure in the drill hole caused by the depth, and the voluminous discharge of groundwater encountered during drilling, decrease the risk of microbial contamination. R-2247 was drilled more than a year prior to our sampling and it had been allowed to flow freely for about a month before it was plugged and the water flow amount was estimated to be around 500 m^3^, equivalent to approximately 175 borehole volumes. The isotopic composition of water from the drill hole R-2250 can be explained by mixing of a saline formation water end-member such as that represented by R-2247 with shallower fresh groundwater, such as the one collected from R-2222 (Figure 6). No evident contribution of drilling water was observed. As the geochemical parameters of both of the R-2250 and R-2247 were also very different from the lake water that was used as a drilling water, this confirms that the sample water originated from the bedrock fractures. In addition, the conditions in the deep bedrock are extreme and very different from those in the water used for drilling. For this reason any possible contaminating agent could survive only as fungal or bacterial spores that may better endure extreme environments. The dominant bacterial sequences found were related to a spore-forming genus in the total community analysis. However, whether this is due to contamination or strong decompression during sampling destroying most live cells is difficult to determine. Nevertheless, the detection of potentially active bacterial, archaeal and fungal taxa suggests that the identified microorganisms were adapted to deep terrestrial subsurface conditions and are able to live in this environment. Yet, evidence indicates that rRNA has some limitations when used as an indicator of current activity as dormant cells could also contain high number of ribosomes (Blazewicz et al., 2013). However, the ITS1 gene region used in this study is present between the 5.8D and 18S units of the mRNA transcript only when the genomic copy of the genes is being actively transcribed. After transcription, the ITS area is quickly spliced out in order for the rRNA units to fold properly and thus is conclusive evidence that the fungi are active and produce ribosomes in the deep biosphere of the Pyhäsalmi mine.

## Conclusions

The presence of deep saline fluids was confirmed in the Pyhäsalmi mine, which supports the previous findings from crystalline shield areas elsewhere in Finland and around the world that these fluids are common below a relatively thin layer of fresh groundwater. The successful geochemical sampling campaign also revealed dissolved gases with compositions clearly distinct from atmospheric air and indicative of long term water-rock interaction. As a mobile phase, dissolved gases such as H_2_ form a potential source of energy for microbial communities in deep terrestrial environments. The microbial high-pressure sampling succeeded only partially as the amount of prokaryotic DNA/RNA collected was especially low. This was probably due to the high-pressure decompression that may have damaged part of the cells and the nucleic acids likely have passed through the filter. The fungal community was discovered to be live and active in the deep subsurface oligotrophic environment, since fungal ITS-sequences were found in the samples. The fungal taxa discovered assist in confirming the fungal core microbiome recently detected from the Fennoscandian Shield bedrock (Sohlberg et al., 2015). The identified prokaryotic diversity was low, however it consisted of species often found from other subsurface environments, but also included taxa such as *Alicyclobacillus* genus and Solimonadaceae that have not been found as dominant bacterial groups in the Fennoscandian Shield bedrock environments before. The majority of the archaeal community was closely-related species to methanogens that use H_2_ and carbon dioxide to produce methane. The geochemical results identified H_2_ and methane as being present, but carbon dioxide was detected at very low levels in two samples. Carbon dioxide may be a limiting factor for these archaea and it may be used rapidly in this highly oligotrophic environment. Conversely, in an alkaline environment (pH over 8), carbon dioxide is present in water as ${\text{HCO}}_{3}^{-}$ and in the Pyhäsalmi drill holes low concentrations of ${\text{HCO}}_{3}^{-}$ was detected. Carbonate minerals, indicative of the precipitation of dissolved carbon, have not been found on fracture surfaces in the studied drill holes. As a result of these experiments we note that a better way to perform microbial sampling in an environment with high water pressure is to maintain high-pressure throughout the filtering and allow for a slow decompression afterwards.

### Conflict of interest statement

The authors declare that the research was conducted in the absence of any commercial or financial relationships that could be construed as a potential conflict of interest.

## References

[B1] Altschul S. F. Gish W. Miller W. Myers E. W. Lipman D. J. (1990). Basic local alignment search tool. J. Mol. Biol. 215, 403–410. 10.1016/S0022-2836(05)80360-2 2231712

[B2] Ballentine C. J. Burgess R. Marty B. (2002). Tracing fluid origin, transport and interaction in the crust. Rev. Mineral. Geochem. 47, 539–614. 10.2138/rmg.2002.47.13

[B3] Bano N. Ruffin S. Ransom B. Hollibaugh J. T. (2004). Phylogenetic composition of Arctic Ocean archaeal assemblages and comparison with Antarctic assemblages. Appl. Environ. Microbiol. 70, 781–789. 10.1128/AEM.70.2.781-789.2004 14766555 PMC348845

[B4] Barns S. M. Fundyga R. E. Jeffries M. W. Pace N. R. (1994). Remarkale archaeal diversity detected in Yellowstone National Park hot spring environment. Proc. Natl. Acad. Sci. U.S.A. 91, 1609–1613. 10.1073/pnas.91.5.1609 7510403 PMC43212

[B5] Blazewicz S. J. Barnard R. L. Daly R. A. Firestone M. K. (2013). Evaluating rRNA as an indicator of microbial activity in environmental communities: limitations and uses. ISME J. 7, 2061–2068. 10.1038/ismej.2013.102 23823491 PMC3806256

[B6] Bomberg M. Itävaara M. (2013). The Diversity of Mirobial Communities in Olkiluoto Groundwater Fracture Zones Charaxterized by DNA and RNA Based 16S rRNA-Targeted 454 Pyro Sequencing and qPCR. Posiva Working Report, 2012-27.

[B7] Bomberg M. Nyyssönen M. Pitkänen P. Lehtinen A. Itävaara M. (2015). Active microbial communities inhabit sulphate-methane interphase in deep bedrock fracture fluids in Olkiluoto, Finland. BioMed. Res. Int. 2015:979530. 10.1155/2015/979530 26425566 PMC4573625

[B8] Bonin A. S. Boone D. R. (2006). The order methanobacteriales, in The Prokaryotes, Vol. 3, eds. Dworkin M. Falkow S. Rosenberg E. Schleifer K.-H. Stackebrandt E. (New York, NY: Springer), 231–243.

[B9] Buée M. Reich M. Murat C. Morin E. Nilsson R. H. Uroz S.. (2009). 454 Pyrosequencing analyses of forest soils reveal an unexpectedly high fungal diversity. New Phytol. 184, 449–456. 10.1111/j.1469-8137.2009.03003.x 19703112

[B10] Caporaso J. G. Kuczynski J. Stombaugh J. Bittinger K. Bushman F. D. Costello E. K.. (2010). QIIME allows analysis of high-throughput community sequencing data. Nat. Methods 7, 335–336. 10.1038/nmeth.f.303 20383131 PMC3156573

[B11] Cathrine S. J. Raghukumar C. (2009). Anaerobic denitrification in fungi from the coastal marine sediments off Goa, India. Mycol. Res. 113, 100–109. 10.1016/j.mycres.2008.08.009 18834939

[B12] Chao A. (1984). Non-parametric estimation of the number of classes in a population. Scand. J. Stat. 11, 265–270.

[B13] Da Costa M. S. Rainey F. A. (2009). Family II. Alicyclobacillaceae fam. Nov, in Bergeys Manual® of Systematic Bacteriology, Vol. 3, eds De Vos P. Garrity G. M. Jones D. Krieg N. R. Ludwig W. Rainey F. A. Schleifer K.-H. Whitman W. B. (New York, NY: Springer), 229.

[B14] Damare S. Raghukumar C. (2008). Fungi and macroaggregation in deep-sea sediments. Microb. Ecol. 56, 168–177. 10.1007/s00248-007-9334-y 17994287

[B15] Davidson M. M. Silver B. J. Onstott T. C. Moser D. P. Gihring T. M. Pratt L. M. (2011). Capture of planktonic microbial diversity in fractures by long-term monitoring of flowing boreholes, Evander Basin, South Africa. Geomicrobiol. J. 28, 275–300. 10.1080/01490451.2010.499928

[B16] DeSantis T. Z. Hugenholtz P. Larsen N. Rojas M. Brodie E. L. Keller K.. (2006). Greengenes, a chimera-checked 16S rRNA gene database and workbench compatible with ARB. Appl. Environ. Microbiol. 72, 5069–5072. 10.1128/AEM.03006-05 16820507 PMC1489311

[B17] Direito S. O. L. Ehrenfreund P. Marees A. Staats M. Foing B. Röling W. F. M. (2011). A wide variety of putative extremophiles and large beta-diversity at the Mars Desert Research Station (Utah). Int. J. Astrobiol. 10, 191–207. 10.1017/S1473550411000012

[B18] Edgar R. C. (2010). Search and clustering orders of magnitude faster than BLAST. Bioinformatics 26, 2460–2461. 10.1093/bioinformatics/btq461 20709691

[B19] Edgcomb V. P. Beaudoin D. Gast R. Biddle J. F. Teske A. (2010). Marine subsurface eukaryotes: the fungal majority. Environ. Microbiol. 13, 172–183. 10.1111/j.1462-2920.2010.02318.x 21199255

[B20] Edwards U. Rogall T. Böcker H. Emde M. Böttger E. C. (1989). Isolation and direct complete nucleotide determination of entire genes. characterization of a gene coding for 16S ribosomal RNA. Nucleic Acids Res. 17, 7843–7853. 10.1093/nar/17.19.7843 2798131 PMC334891

[B21] Ekendahl S. O'Neill A. H. Thomsson E. Pedersen K. (2003). Characterisation of yeasts isolated from deep igneous rock aquifers of the Fennoscandian shield. Microb. Ecol. 46, 416–428. 10.1007/s00248-003-2008-5 14502418

[B22] Eydal H. S. C. Jägevall S. Hermansson M. Pedersen K. (2009). Bacteriophage lytic to *Desulfovibrio aespoeensis* isolated from deep groundwater. ISME J. 3, 1139–1147. 10.1038/ismej.2009.66 19516280

[B23] Fell J. W. Kurtzman C. P. Tallman A. S. Buck J. D. (1988). Rhodosporidium fluviale sp. nov., a homokaryotic red yeast from a subtropical brackish environment. Mycologia 80, 560–564. 10.2307/3807858

[B24] Frape S. K. Blyth A. Blomqvist R. McNutt R. H. Gascoyne M. (2003). Deep fluids in the continents: II. Crystalline rocks. Treatise Geochem. 5, 541–580. 10.1016/B0-08-043751-6/05086-6

[B25] Fredrickson J. K. Balkwill D. L. (2006). Geomicrobial processes and biodiversity in the deep terrestrial subsurface. Geomicrobiol. J. 23, 345–356. 10.1080/01490450600875571

[B26] Frostegård Å. Courtois S. Ramisse V. Clerc S. Bernillon D. Le Gall F.. (1999). Quantification of bias related to the extraction of DNA directly from soils. Appl. Environ. Microbiol. 65, 5409–5420. 10583997 10.1128/aem.65.12.5409-5420.1999 PMC91737

[B27] Gardes M. Bruns T. D. (1993). ITS primers with enhanced specificity for basidiomycetes—application to the identification of mycorrhizae and rusts. Mol. Ecol. 2, 113–118. 10.1111/j.1365-294X.1993.tb00005.x 8180733

[B28] Griebler C. Lueders T. (2009). Microbial biodiversity in groundwater ecosystems. Freshw. Biol. 54, 649–677. 10.1111/j.1365-2427.2008.02013.x

[B29] Großkopf R. Stubner S. Liesack W. (1998). Novel euryarchaeotal lineages detected on rice roots and in the anoxic bulk soil of flooded rice microcosms. Appl. Environ. Microbiol. 64, 4983–4989. 9835592 10.1128/aem.64.12.4983-4989.1998 PMC90952

[B30] Hallbeck L. Pedersen K. (2012). Culture-dependent comparison of microbial diversity in deep granitic groundwater from two sites considered for a swedish final repository of spent nuclear fuel. FEMS Microbiol. Ecol. 81, 66–77. 10.1111/j.1574-6941.2011.01281.x 22188407

[B31] Hatanpää E. Manninen P. Apilo S. (2005). Representativity of Gas Samples Taken with the Pressurized Water Sampling System (PAVE) 1995–2004. Posiva Working Report, 5005-55.

[B32] Haveman S. A. Pedersen K. Ruotsalainen P. (1999). Distribution and metabolic diversity of microorganisms in deep igneous rock aquifers of Finland. Geomicrobiol. J. 16, 277–294. 10.1080/014904599270541

[B33] Hemmingsen B. B. Hemmingsen E. A. (1980). Rupture of the cell envelope by induced intracellular gas phase expansion in gas vacuolate bacteria. J. Baceriol. 143, 841–846. 7204336 10.1128/jb.143.2.841-846.1980 PMC294375

[B34] Hirose T. Kawagucci S. Suzuki K. (2011). Mechanoradical H_2_ generation during simulated faulting: Implications for an earthquake-driven subsurface biosphere. Geophys. Res. Lett. 38, L17303 10.1029/2011GL048850

[B35] Hoehler T. M. Jørgensen B. B. (2013). Microbial life under extreme energy limitation. Nat. Rev. 11, 83–94. 10.1038/nrmicro2939 23321532

[B36] Hurt R. A. Jr. Robeson M. S. II. Shakya M. Moberly J. G. Vishnivetskaya T. A. Gu B.. (2014). Improved yield of high molecular weight DNA coincides with increased microbial diversity access from iron oxide cemented sub-surface clay environments. PLoS ONE 9:e102826. 10.1371/journal.pone.0102826 25033199 PMC4102596

[B37] Imperio T. Viti C. Marri L. (2008). *Alicyclobacillus pohliae* sp. nov., a thermophilic, endospore-forming bacterium isolated from geothermal soil of the north-west slope of Mount Melbourne (Antarctica). Int. J. Syst. Ecol. Microbiol. 58, 221–225. 10.1099/ijs.0.65092-0 18175712

[B38] Itävaara M. Nyyssönen M. Kapanen A. Nousiainen A. Ahonen L. Kukkonen I. (2011). Characterization of bacterial diversity down to a depth of 1500 m in the Outokumpu deep borehole, Fennoscandian shield. FEMS Microbiol. Ecol. 77, 295–309. 10.1111/j.1574-6941.2011.01111.x 21488910

[B39] Itävaara M. Vehkomäki M. L. Nousiainen A. (2008). Sulphate-Reducing Bacteria in Ground Water Samples from Olkiluoto - Analyzed by Quantitative PCR. Posiva Working Report, 2008-82.

[B40] Kärki A. Laajoki K. Luukas J. (1993). Major Palaeoproterozoic shear zones of the central Fennoscandian Shield. Precambrian Res. 64, 207–223. 10.1016/0301-9268(93)90077-F

[B41] Kellogg C. A. Griffin D. W. (2006). Aerobiology and the global transport of desert dust. Trends Ecol. Evol. 21, 638–644. 10.1016/j.tree.2006.07.004 16843565

[B42] Keppner R. L. Jr. Pratt J. R. (1994). Use of fluorochromes for direct enumeration of total bacteria in environmental samples: past and present. Microbiol. Rev. 58, 603–615. 7854248 10.1128/mr.58.4.603-615.1994 PMC372983

[B43] Kietäväinen R. Ahonen L. Kukkonen I. T. Hendriksson N. Nyyssönen M. Itävaara M. (2013). Characterisation and isotopic evolution of saline waters of the Outokumpu Deep Drill Hole, Finland - implications for water origin and deep terrestrial biosphere. Appl. Geochem. 32, 37–51. 10.1016/j.apgeochem.2012.10.013

[B44] Kietäväinen R. Ahonen L. Kukkonen I. T. Niedermann S. Wiersberg T. (2014). Noble gas residence times of saline waters within crystalline bedrock, Outokumpu Deep Drill Hole, Finland. Geochim. Cosmochim. Acta 145, 159–174. 10.1016/j.gca.2014.09.012

[B45] Kita I. Matsuo S. Wakita H. (1982). H_2_ generation by reaction between H_2_O and crushed rock: an experimental study on H_2_ degassing from the active fault zone. J. Geophys. Res. 87, 10789–10795. 10.1029/JB087iB13p10789

[B46] Kõljalg U. Nilsson R. H. Abarenkov K. Tedersoo L. Taylor A. F. S. Bahram M.. (2013). Towards a unified paradigm for sequence-based identification of fungi. Mol. Ecol. 22, 5271–5277. 10.1111/mec.12481 24112409

[B47] Kormas K. A. Smith D. C. Edgcomb V. Teske A. (2003). Molecular analysis of deep subsurface microbial communities in Nankai Trough sediments (ODP Leg190, Site 1176). FEMS Microbiol. Ecol. 45, 115–125. 10.1016/S0168-6496(03)00128-4 19719622

[B48] Kortelainen N. (2007). Isotopic Fingerprints in Surficial Waters: Stable Isotope Methods Applied in Hydrogeological Studies. Geological Survey of Finland, Espoo.

[B49] Kotelnikova S. Pedersen K. (1998). Distribution and activity of methanogens and homoacetogens in deep granitic aquifers at Äspö hard rock laboratory, Sweden. FEMS Microbiol. Ecol. 26, 121–134.

[B50] Kubo Y. Mizuguchi Y. Inagaki F. Yamamoto K. (2014). A new hybrid pressure-coring system for the drilling vessel. Chikyu. Sci. Dril. 17, 37–43. 10.5194/sd-17-37-2014

[B51] Kukkonen I. (1989). Terrestrial Heat Flow in Finland, the Central Fennoscandian Shield. Geological Survey of Finland, Nuclear Waste Disposal Research, Report YST-68. Available online at: http://tupa.gtk.fi/julkaisu/ydinjate/yst_068.pdf

[B52] Kurakov A. V. Lavrent'Ev R. B. Nechitailo T. Y. Golyshin P. N. Zvyagintsev D. G. (2008). Diversity of facultatively anaerobic microscopic mycelial fungi in soils. Microbiology 77, 90–98. 10.1134/S002626170801013X 18365728

[B53] Laine E. Luukas J. Mäki T. Kousa J. Ruotsalainen A. Suppala I. (2015). The Vihanti-Pyhäsalmi area, in 3d, 4d and Predictive Modelling of Mineral Belts: European Resources Under Cover, ed Weihed P. (New York, NY: Springer), 123–144.

[B54] Lin L. Hall J. Lippmann-Pipke J. Ward J. A. Sherwood Lollar B. DeFlaun M. (2005a). Radiolytic H_2_ in continental crust: nuclear power for deep subsurface microbial communities. Geochem. Geophys. Geosyst. 6, Q07003 10.1016/j.gca.2004.07.032

[B55] Lin L.-H. Slater G. F. Sherwood Lollar B. Lacrampe-Couloume G. Onstott T. C. (2005b). The yield and isotopic composition of radiolytic H_2_, a potential energy source for the deep subsurface biosphere. Geochim. Cosmochim. Acta 69, 893–903. 10.1016/j.gca.2004.07.032

[B56] Lorenz R. Molitoris H. P. (1997). Cultivation of fungi under simulated deep sea conditions. Mycol. Res. 101, 1355–1365.

[B57] Mayhew L. E. Ellison E. T. McCollom T. M. Trainor T. P. Templeton A. S. (2013). Hydrogen generation from low-temperature water-rock reactions. Nat. Geosci. 6, 478–484. 10.1038/ngeo1825

[B58] McMahon S. Parnell J. (2013). Weighing the deep continental biosphere. FEMS Microbial. Ecol. 87, 113–120. 10.1111/1574-6941.12196 23991863

[B59] Miettinen H. Bomberg M. Nyyssönen M. Salavirta H. Sohlberg E. Vikman M. (2015). The Diversity Of Microbial Communities in Olkiluoto Bedrock Groundwaters 2009-2013. Posiva Working Report, 2015-12.

[B60] Moser D. P. Gihring T. M. Brockman F. J. Fredrickson J. K. Balkwill D. L. Dollhopf M. E.. (2005). Desulfotomaculum and Methanobacterium spp. dominate a 4- to 5-kilometer-deep fault. Appl. Environ. Microbiol. 71, 8773–8783. 10.1128/AEM.71.12.8773-8783.2005 16332873 PMC1317344

[B61] Moser D. P. Onstott T. C. Fredrickson J. K. Brockman F. J. Balkwill D. L. Drake G. R. (2003). Temporal shifts in microbial community structure and geochemistry of an ultradeep South African gold mine borehole. Geomicrobiol. J. 20, 1–32. 10.1080/713851170

[B62] Muyzer G. de Waal E. C. Uitterlinden A. G. (1993). Profiling of complex microbial populations by denaturing gradient gel electrophoresis analysis of polymerase chain reaction-amplified genes coding 16S rRNA. Appl. Environ. Microbiol. 59, 695–700. 7683183 10.1128/aem.59.3.695-700.1993 PMC202176

[B63] Nagahama T. Hamamoto M. Horikoshi K. (2006). Rhodotorula pacifica sp. nov., a novel yeast species from sediment collected on the deep-sea floor of the north-west Pacific Ocean. Int. J. Syst. Evol. Microbiol. 56, 295–299. 10.1099/ijs.0.63584-0 16403901

[B64] Nagahama T. Hamamoto M. Nakase T. Takami H. Horikoshi K. (2001). Distribution and identification of red yeasts in deep-sea environments around the northwest Pacific Ocean. Antonie Van Leeuwenhoek 80, 101–110. 10.1023/A:1012270503751 11759043

[B65] Nagano Y. Nagahama T. Hatada Y. Nunoura T. Takami H. Miyazaki J. (2010). Fungal diversity in deep-sea sediments–the presence of novel fungal groups. Fungal Ecol. 3, 316–325. 10.1016/j.funeco.2010.01.002

[B66] Nguyen T. H. Elimelech M. (2007). Plasmid DNA adsorption on silica: kinetics and conformational changes in monovalent and divalent salts. Biomacromolecules 8, 24–32. 10.1021/bm0603948 17206784

[B67] Nübel U. Wngelen B. Felske A. Snaidr J. Wieshuber A. Amann R. I. (1996). Sequence heterogeneities of genes encoding 16S rRNAs in *Paenibacillus polymyxa* detected by temperature gradient gel electrophoresis. J. Bacteriol. 178, 5636–5643. 8824607 10.1128/jb.178.19.5636-5643.1996 PMC178401

[B68] Nurmi P. A. Kukkonen I. T. Lahermo P. W. (1988). Geochemistry and origin of saline groundwaters in the Fennoscandian shield. Appl. Geochem. 3, 185–203. 10.1016/0883-2927(88)90007-8

[B69] Nyyssönen M. Bomberg M. Kapanen A. Nousiainen A. Pitkänen P. Itävaara M. (2012). Methanogenic and sulphate-reducing microbial communities in deep groundwater of crystalline rock fractures in Olkiluoto, Finland. Geomicrobiol. J. 29, 863–878. 10.1080/01490451.2011.635759

[B70] Nyyssönen M. Hultman J. Ahonen L. Kukkonen I. Paulin L. Laine P.. (2014). Taxonomically and functionally diverse microbial communities in deep crystalline rocks of the Fennoscandian shield. ISME J. 8, 126–138. 10.1038/ismej.2013.125 23949662 PMC3869007

[B71] Onstott T. C. McGown D. J. Bakermans C. Ruskeeniemi T. Ahonen L. Telling J.. (2009). Microbial communities in subpermafrost saline fracture water at the Lupin Au Mine, Nunavut, Canada. Microb. Ecol. 58, 786–807. 10.1007/s00248-009-9553-5 19568805

[B72] Orsi W. Biddle J. F. Edgcomb V. (2013). Deep sequencing of subseafloor eukaryotic rRNA reveals active fungi across marine subsurface provinces. PLoS ONE 8:e56335. 10.1371/journal.pone.0056335 23418556 PMC3572030

[B73] Paget E. Monrozier L. J. Simonet P. (1992). Adsorption of DNA on clay minerals: protection against DNaseI and influence on gene transfer. FEMS Microbiol. Lett. 97, 31–39. 10.1111/j.1574-6968.1992.tb05435.x

[B74] Park C. B. Clark D. S. (2002). Rupture of the cell envelope by decompression of the deep-sea methanogen *Methanococcus jannaschii*. Appl. Environ. Microbiol. 68, 1458–1463. 10.1128/AEM.68.3.1458-1463.2002 11872502 PMC123755

[B75] Pedersen K. (1987). Preliminary Investigations of Deep Ground Water Microbiology in Swedish Granitic Rock. SKB Technical Report 88-01, Swedish nuclear fuel and waste management Co., Stockholm.

[B76] Purkamo L. Bomberg M. Nyyssönen M. Kukkonen I. Ahonen L. Kietäväinen R.. (2013). Dissecting the deep biosphere: retrieving authentic microbial communities from packer-isolated deep crystalline bedrock fracture zones. FEMS Microbiol. Ecol. 85, 324–337. 10.1111/1574-6941.12126 23560597

[B77] Raghukumar C. (ed.). (2012). Biology of Marine Fungi, Vol. 53 Heidelberg: Springer 10.1007/978-3-642-23342-5_5

[B78] Ragon M. Van Driessche A. E. S. García-Ruíz J. M. Moreira D. López-García P. (2013). Microbial diversity in the deep-subsurface hydrothermal aquifer feeding the giant gypsum crystal-bearing Naica Mine, Mexico. Front. Microbiol. 4:37. 10.3389/fmicb.2013.00037 23508882 PMC3589807

[B79] Reed D. W. Fujita Y. Delwiche M. E. Blackwelder D. B. Sheridan P. P. Uchida T.. (2002). Microbial communities from methane hydrate-bearing deep marine sediments in a Forearc Basin. Appl. Environ. Microbiol. 68, 3759–3770. 10.1128/AEM.68.8.3759-3770.2002 12147470 PMC124055

[B80] Regenspurg S. Wiersberg T. Brandt W. Huenges E. Saadat A. Schmidt K. (2010). Geochemical properties of saline geothermal fluids from the *in-situ* geothermal laboratory GroßSchönebeck (Germany). Chem. Erde 70, 3–12. 10.1016/j.chemer.2010.05.002

[B81] Schloss P. D. Westcott S. L. Ryabin T. Hall J. R. Hartmann M. Hollister E. B.. (2009). Introducing mothur: Open-source, platform-independent, community-supported software for describing and comparing microbial communities. Appl. Environ. Microbiol. 75, 7537–7541. 10.1128/AEM.01541-09 19801464 PMC2786419

[B82] Sherwood Lollar B. Vogelsonger K. Lin L.-H. Lacrampe-Couloume G. Telling J. Abrajano T. A.. (2007). Hydrogeologic controls on episodic H_2_ release from Precambrian fractured rocks - Energy for deep subsurface life on Earth and Mars. Astrobiology 7, 971–986. 10.1089/ast.2006.0096 18163873

[B83] Singh P. Raghukumar C. Verma P. Shouche Y. (2012). Assessment of fungal diversity in deep-sea sediments by multiple primer approach. World J. Microbiol. Biotechnol. 28, 659–667. 10.1007/s11274-011-0859-3 22806861

[B84] Sohlberg E. Bomberg M. Miettinen H. Nyyssönen M. Salavirta H. Vikman M.. (2015). Revealing the unexplored fungal communities in deep groundwater of crystalline bedrock fracture zones in Olkiluoto, Finland. Front. Microbiol. 6:573. 10.3389/fmicb.2015.00573 26106376 PMC4460562

[B85] Stahl D. A. Amann R. (1991). Development and application of nucleic acid probes, in Nucleic Acid Techniques in Bacterial Systematics, eds Stackebrandt E. Goodfellow M. (Chichster: John Wiley & Sons), 205–248.

[B86] Stotler R. L. Frape S. K. Ruskeeniemi T. Ahonen L. Onstott T. C. Hobbs M. Y. (2009). Hydrogeochemistry of groundwaters in and below the base of thick permafrost at Lupin, Nunavut, Canada. J. Hydrol. 373, 80–95. 10.1016/j.jhydrol.2009.04.013

[B87] Takai K. Moser D. P. DeFlaun M. Onstott T. C. Fredrickson J. K. (2001). Archaeal diversity in waters from deep South African gold mines. Appl. Environ. Microbiol. 67, 5750–5760. 10.1128/AEM.67.21.5750-5760.2001 11722932 PMC93369

[B88] Trinchero P. Delos A. Molinero J. Dentz M. Pitkänen P. (2014). Understanding and modelling dissolved gas transport in the bedrock of three Fennoscandian sites. J. Hydrol. 512, 506–517. 10.1016/j.jhydrol.2014.03.011

[B89] USGS (2014). PHREEQC. Computer codes, United States Geological Survey Available online at: http://wwwbrr.cr.usgs.gov/projects/GWC_coupled/phreeqc/

[B90] Wang Q. Carrity G. M. Tiedje J. M. Cole J. R. (2007). Naïve bayesian classifier for rapid assignment of rRNA Sequences into the new bacterial taxonomy. Appl. Environ. Microbiol. 73, 5261. 10.1128/AEM.00062-07 17586664 PMC1950982

[B91] Weisburg W. G. Barns S. M. Pelletier D. A. Lane D. J. (1991). 16S ribosomal DNA amplification for phylogenetic study. J. Bacteriol. 173, 697–703. 1987160 10.1128/jb.173.2.697-703.1991 PMC207061

[B92] White T. J. Bruns T. Lee S. Taylor J. (1990). Amplification and direct sequencing of fungal ribosomal RNA genes for phylogenetics, in PCR Protocols: A Guide to Methods and Applications, ed Innis M. (San Diego, CA: AcademicPress), 315–322.

[B93] Yashiro Y. Sakai S. Ehara M. Miyazaki M. Yamaguchi T. Imachi H. (2011). Methanoregula formicica sp. nov., a methane-producing archaeon isolated from methanogenic sludge. Int. J. Syst. Evol. Microbiol. 61, 53–59. 10.1099/ijs.0.014811-0 19667393

[B94] Zhou Y. Lai R. Li W.-J. (2014). The family solimonadaceae, in The Prokaryotes, eds Rosenberg E. De Long E. F. Lory S. Stackebrandt E. Thompson F. (Heidelberg; Berlin: Springer), 627–638.

