# Supplementary material for: Microbiome composition and geochemical characteristics of deep subsurface high-pressure environment, Pyhäsalmi mine Finland
Source: Front Microbiol. 2015 Oct 30;6:1203. doi: 10.3389/fmicb.2015.01203 (PMC4626562; doi:10.3389/fmicb.2015.01203)
Supplement: Supplementary file 1 [file Table1.PDF]

Supplement Table 1. Geochemical characteristics of drill hole fluids from the Pyhäsalmi mine, central Finland.

**Sample code**	**Drill hole**	**Sampling date**	**Depth mbsl**[1]	**pH**	**EC**[2] **mS cm^{-1}**	**Alk.**[3] **mmol L^{-1}**	**TOC**[4] **mmol L^{-1}**	**DOC**[5] **mmol L^{-1}**	**TDS**[6] **g L^{-1}**	**δ^2H_{H2O} ‰ VSMOW**	**$\delta^{18}O_{H2O}$ ‰ VSMOW**	**Ag µmol L^{-1}**	**Al µmol L^{-1}**
PY-2	R-2222	27/5/2013	850	7.5	4.92				2.34	-96.8	-13.44	0.0002	0.13
PY-3	PH-101	28/5/2013	1080	7.0	46.6	0.05			24.4	-95.5	-13.68	0.0013	0.37
PYH-4	R-2250	14/8/2013	1350	8.8	34.0	0.09	0.117	0.085	17.7	-86.2	-13.28	0.0006	0.57
PY-1	R-2227	27/5/2013	1430	8.4		0.13			28.8	-88.9	-13.21	0.0017	0.29
PYH-3	R-2227	13/8/2013	1430	8.8	46.6				27.9			0.0009	0.50
PYS-2	R-2227	11/6/2014	1430	8.9	44.9	0.07			25.7	-91.2	-13.70	0.0008	0.21
PY-4	R-2229	28/5/2013	1430	7.8	57.4	0.07			34.3	-86.8	-13.06	0.0016	0.18
PYH-2	R-2229	13/8/2013	1430	8.5	57.0	0.07	0.057	0.036	34.1	-87.3	-13.28	0.0015	0.42
PY-5	R-2247	28/5/2013	1430	8.1	105.5	0.11			72.4	-69.8	-11.96	0.0030	0.25
PYH-1	R-2247	13/8/2013	1430	8.6	103.3	0.09	0.092	0.066	76.2	-70.7	-12.52	0.0032	0.18
PYS-1	R-2247	11/6/2014	1430	8.7	102.0	0.08			66.9	-70.2	-12.27	0.0025	0.44

[1]mbsl = Depth of the borehole collar at metres below surface level.

[2]EC = electrical conductivity

[3]Alk. = alkalinity

[4]TOC = total organic carbon

[5]DOC = dissolved organic carbon

[6]TDS = total dissolved solids

Supplement Table 1. continues

Sample code	As μmol L^{-1}	B mmol L^{-1}	Ba mmol L^{-1}	Bi μmol L^{-1}	Ca mmol L^{-1}	Cd μmol L^{-1}	Co μmol L^{-1}	Cr μmol L^{-1}	Cu μmol L^{-1}	Fe mmol L^{-1}	K mmol L^{-1}	Li mmol L^{-1}	Mg mmol L^{-1}
PY-2	0.002	0.016	0.00015	0.00010	13	bdl	0.003	bdl	0.05	bdl	0.07	0.013	0.14
PY-3	0.020	0.019	0.002	0.0026	168	0.0008	0.048	0.016	0.11	0.0011	0.21	0.11	0.92
PYH-4	0.033	0.067	0.0008	0.0004	119	0.0007	0.036	0.013	0.008	0.0006	0.14	0.03	0.04
PY-1	0.026	0.051	0.003	0.0041	192	0.0004	0.053	0.037	0.13	bdl	0.34	0.12	0.11
PYH-3	0.043	0.049	0.003	0.0007	198	0.0011	0.063	0.028	0.012	0.0009	0.32	0.02	0.08
PYS-2	0.051	0.045	0.002	bdl[1]	174	0.0006	0.018	0.017	0.05	bdl	0.39	0.012	0.07
PY-4	0.032	0.057	0.005	0.0032	222	0.0008	0.064	0.022	0.10	0.0009	0.47	0.14	0.22
PYH-2	0.050	0.058	0.005	0.0011	249	0.0017	0.086	0.034	0.013	0.0012	0.47	0.016	0.19
PY-5	0.09	0.072	0.007	0.0048	551	0.0023	0.15	0.049	0.15	0.0020	0.82	0.35	0.17
PYH-1	0.12	0.082	0.008	0.0010	565	0.0023	0.21	0.083	0.03	0.0024	0.83	0.08	0.14
PYS-1	0.17	0.072	0.006	0.0043	488	0.0014	0.058	0.040	0.20	bdl	0.97	0.07	0.13

[1]bdl = below detection limit

Supplement Table 1. continues

**Sample code**	**Mn mmol L^{-1}**	**Mo µmol L^{-1}**	**Na mmol L^{-1}**	**Ni µmol L^{-1}**	**P mmol L^{-1}**	**Pb µmol L^{-1}**	**Rb µmol L^{-1}**	**S_{tot} mmol L^{-1}**	**Sb µmol L^{-1}**	**Se µmol L^{-1}**	**Si mmol L^{-1}**	**Sr mmol L^{-1}**	**U µmol L^{-1}**	**V µmol L^{-1}**
PY-2	0.0003	0.024	17	bdl[1]	0.0007	0.0012	0.17	0.88	0.0014	bdl	0.33	0.06	0.0072	0.005
PY-3	0.0021	0.017	107	0.12	0.0013	0.0004	0.62	0.34	0.0019	0.013	0.14	0.99	0.00004	0.034
PYH-4	0.0004	0.018	112	0.08	0.003	0.0004	0.65	1.16	bdl	0.018	0.18	0.61	0.0012	0.029
PY-1	0.0004	0.063	134	0.16	0.0014	0.0007	1.07	1.01	0.0025	0.025	0.13	1.12	0.0001	0.038
PYH-3	0.0003	0.039	144	0.14	0.003	0.0003	0.94	1.14	0.013	0.030	0.12	1.16	0.0007	0.046
PYS-2	0.0002	0.020	137	0.16	0.003	0.0019	0.79	1.20	0.0011	0.127	0.13	1.10	0.00004	0.092
PY-4	0.0011	0.026	157	0.16	0.002	0.0004	1.74	0.70	0.0026	0.016	0.17	1.31	0.00004	0.049
PYH-2	0.0012	0.041	183	0.18	0.003	0.0005	1.76	0.71	0.0014	0.036	0.15	1.48	0.0015	0.059
PY-5	0.0025	0.016	332	0.36	0.002	0.0004	3.98	1.31	0.0052	0.028	0.16	3.41	0.0001	0.12
PYH-1	0.0025	0.047	324	0.40	0.005	0.0005	3.97	1.23	0.0047	0.066	0.14	3.06	0.0016	0.13
PYS-1	0.0016	0.031	320	0.39	0.004	0.0008	3.35	1.30	0.0045	0.137	0.10	3.02	0.00006	0.23

[1]bdl = below detection limit

Supplement Table 1. continues

Sample code	Th µmol L-1	Zn µmol L-1	Br mmol L-1	Cl mmol L-1	I mmol L-1	SO_4 mmol L-1	Sulfide mmol L-1
PY-2	bdl[1]	0.05	bdl	39	0.0006		
PY-3	bdl	0.04	3.24	417	0.004		
PYH-4	0.00004	0.06	2.17	285	0.003	1.23	0.057
PY-1	0.00004	0.50	3.33	496	0.004		
PYH-3	0.00009	0.07	2.95	460	0.004	2.04	
PYS-2	bdl	0.05	2.43	426	0.010	1.19	0.0042
PY-4	0.00004	0.04	4.09	601	0.005		
PYH-2	0.00004	0.09	5.38	544	0.005	1.10	0.0019
PY-5	0.00004	0.04	7.83	1173	0.009		
PYH-1	0.00013	0.04	7.48	1272	0.010	1.78	0.0023
PYS-1	0.00004	0.05	6.65	1097	0.019	1.58	0.0020

[1]bdl = below detection limit
