# Supplementary material for: Microbiome composition and geochemical characteristics of deep subsurface high-pressure environment, Pyhäsalmi mine Finland
Source: Front Microbiol. 2015 Oct 30;6:1203. doi: 10.3389/fmicb.2015.01203 (PMC4626562; doi:10.3389/fmicb.2015.01203)
Supplement: Supplementary file 2 [file Table2.PDF]

Supplement Table 2. Gas compositions in the drill hole fluids from the Pyhäsalmi mine.

| **Sample code** | **Drill hole** | **Sampling date** | **Gas method**[1] | **Ar mL L$^{-1}$** | **$O_2$ mL L$^{-1}$** | **$N_2$ mL L$^{-1}$** | **$CO_2$ mL L$^{-1}$** | **He mL L$^{-1}$** | **$CH_4$ mL L$^{-1}$** | **$H_2$ mL L$^{-1}$** | **$C_2H_6$ mL L$^{-1}$** | **Iso-butane mL L$^{-1}$** | **N-butane mL L$^{-1}$** | **N-pentane mL L$^{-1}$** |
|---|---|---|---|---|---|---|---|---|---|---|---|---|---|---|
| PY-3 | PH-101 | 28/5/2013 | FF/G | 0.22 | 0.08 | 15.5 | bdl | 5.6 | 0.12 | bdl | 0.010 | | | |
| PYH-4 | R-2250 | 14/8/2013 | FF/G | 0.40 | 0.19 | 25.8 | bdl | 19.0 | 13.5 | 0.74 | 0.080 | | | |
| PY-1 | R-2227 | 27/5/2013 | FF/G | 0.47 | 0.15 | 33.4 | bdl | 11.4 | 0.10 | 0.18 | 0.022 | | | |
| PY-1/II | R-2227 | 28/5/2013 | FF/G | 0.47 | 0.13 | 33.5 | bdl | 11.4 | 0.11 | 0.18 | 0.022 | | | |
| PYH-3 | R-2227 | 13/8/2013 | FF/G | 0.45 | 0.15 | 32.0 | bdl | 10.8 | 0.12 | 0.18 | 0.018 | | | |
| PYS-2 | R-2227 | 11/6/2014 | *PF/C*[2] | *0.40* | *5.0* | *34.7* | *bdl* | *5.0* | *0.05* | *0.11* | *0.006* | | | |
| PYS-2B | R-2227 | 11/6/2014 | FF/I | 0.42 | 0.12 | 33.4 | bdl | 12.3 | 0.14 | 0.01 | 0.019 | bdl | 0.00009 | bdl |
| PYS-2C | R-2227 | 11/6/2014 | FF/I | 0.51 | 0.23 | 32.8 | bdl | 11.3 | 0.13 | 0.24 | 0.017 | | | |
| PYS-2D | R-2227 | 11/6/2014 | FF/G | 0.46 | 0.14 | 32.9 | bdl | 11.3 | 0.13 | 0.25 | 0.018 | | | |
| PY-4 | R-2229 | 28/5/2013 | FF/G | 0.58 | 0.17 | 42.6 | bdl | 13.1 | 0.11 | 0.08 | 0.040 | | | |
| PYH-2 | R-2229 | 13/8/2013 | FF/G | 0.58 | 0.26 | 48.1 | bdl | 12.7 | 0.12 | bdl | 0.037 | | | |
| PYH-2A | R-2229 | 13/8/2013 | PF/C | 0.62 | 0.49 | 44.3 | 0.53 | 15.8 | 0.11 | bdl | 0.037 | | | |
| PY-5 | R-2247 | 28/5/2013 | FF/G | 0.86 | 0.30 | 58.2 | bdl | 21.4 | 2.32 | 0.05 | 0.38 | | | |
| PYH-1 | R-2247 | 13/8/2013 | FF/G | 0.58 | 0.31 | 38.8 | bdl | 14.5 | 1.56 | 0.10 | 0.24 | | | |
| PYS-1 | R-2247 | 11/6/2014 | PF/C | 1.10 | bdl[3] | 64.5 | bdl | 36.0 | 3.56 | 10.3 | 0.39 | | | |
| PYS-1A | R-2247 | 11/6/2014 | FF/I | 1.16 | 0.72 | 73.7 | bdl | 27.2 | 3.80 | 8.67 | 0.44 | | | |
| PYS-1B | R-2247 | 11/6/2014 | FF/I+ | 1.02 | 0.25 | 75.2 | 0.012 | 29.1 | 4.08 | 8.48 | 0.50 | 0.00024 | 0.0012 | 0.00012 |
| PYS-1C | R-2247 | 11/6/2014 | FF/I | 0.92 | 0.26 | 75.3 | bdl | 29.1 | 4.10 | 8.51 | 0.50 | 0.00024 | 0.0012 | 0.00012 |
| PYS-1D | R-2247 | 11/6/2014 | FF/G | 1.08 | 0.37 | 73.6 | bdl | 27.5 | 3.92 | 8.79 | 0.45 | | | |

[1]FF/G = from free flowing fluid into glass bottle (Schott); FF/I = from free flowing fluid injected into glass bottle (Laborexin) + = $HgCl_2$ added; PF/C = from pressurized fluid into stainless steel cylinder
[2]Likely contaminated with air
[3]bdl = below detection limit
