# Supplementary material for: Microbiome composition and geochemical characteristics of deep subsurface high-pressure environment, Pyhäsalmi mine Finland
Source: Front Microbiol. 2015 Oct 30;6:1203. doi: 10.3389/fmicb.2015.01203 (PMC4626562; doi:10.3389/fmicb.2015.01203)
Supplement: Supplementary file 3 [file Table3.PDF]

Supplement Table 3. General sequence statistics of bacterial and archaeal 16SrDNA and 16S rRNA sequences and fungal ITS sequences in the drill hole fluids from the Pyhäsalmi mine, central Finland. OTUs are reported at 97% identity and the alpha diversity of the sequence data is estimated.

Sample target	Sample code	Drill hole	Sampling date	Depth mbsl[1]	Sample type	Number of sequences	OTUs (97%)	Chao1	% diversity obtained
Bacteria	PYH-4	R-2250	14/8/2013	1350	DNA	304	18	18	100
	PYH-4	R-2250	14/8/2013	1350	RNA	946	45	45	100
	PYH-1	R-2247	13/8/2013	1430	DNA	137	19	26	73
	PYH-1	R-2247	13/8/2013	1430	RNA	23	6	6	100
Archaea	PYH-4	R-2250	14/8/2013	1350	DNA	3485	4	4	100
	PYH-4	R-2250	14/8/2013	1350	RNA	3044	6	6	100
	PYH-1	R-2247	13/8/2013	1430	DNA	4	2	2	100
	PYH-1	R-2247	13/8/2013	1430	RNA	1	1	1	100
Fungi	PYH-4	R-2250	14/8/2013	1350	DNA	11730	28	37	76
	PYH-4	R-2250	14/8/2013	1350	RNA	17514	13	15	87
	PYH-1	R-2247	13/8/2013	1430	DNA	32341	77	77	100
	PYH-1	R-2247	13/8/2013	1430	RNA	13626	13	18	72

[1]mbsl = Depth of the borehole collar at metres below surface level.
