# Supplementary figures and images for: Microbiome composition and geochemical characteristics of deep subsurface high-pressure environment, Pyhäsalmi mine Finland
Source: Front Microbiol. 2015 Oct 30;6:1203. doi: 10.3389/fmicb.2015.01203 (PMC4626562; doi:10.3389/fmicb.2015.01203)

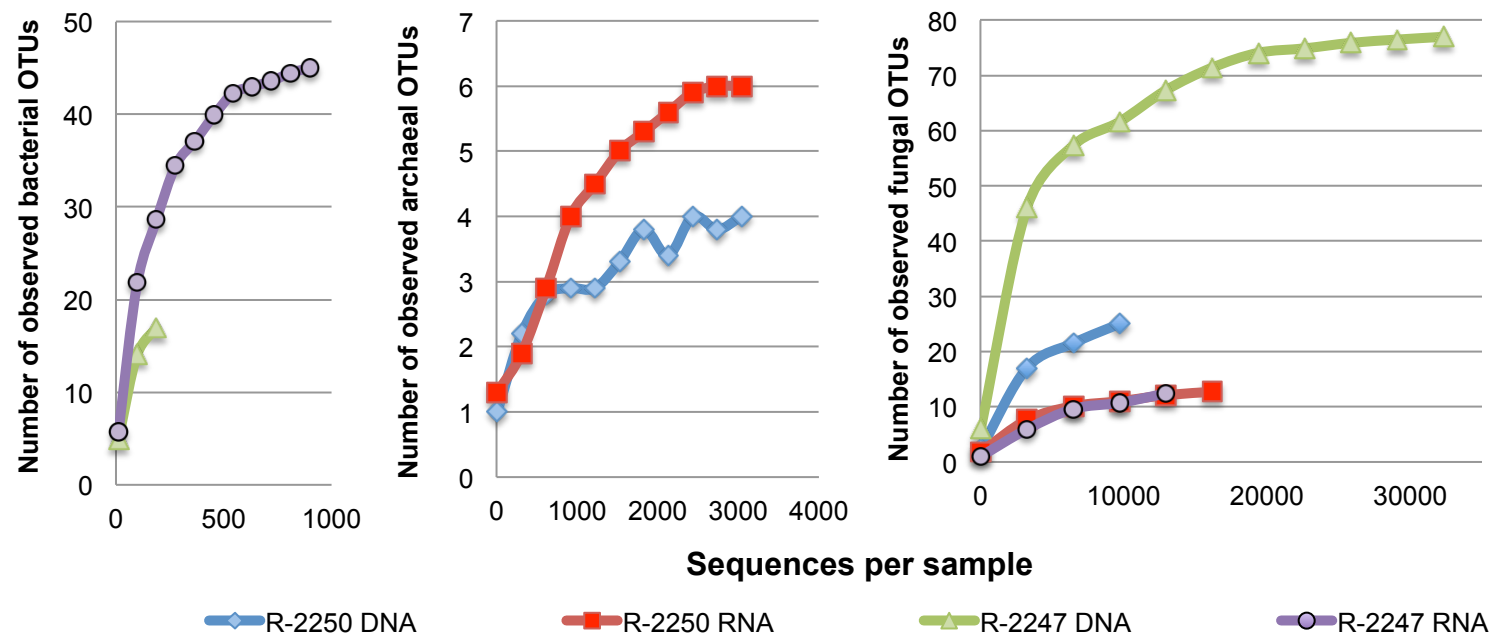

Image 1. Rarefaction curves from samples with more than 300 sequence reads.

Supplement: Supplementary file 4 [file Image1.PDF]
